# Rejuvenation of Bone Marrow Mesenchymal Stem Cells: Mechanisms and Their Application in Senile Osteoporosis Treatment

**DOI:** 10.3390/biom15020276

**Published:** 2025-02-13

**Authors:** Rui-Chuan Tian, Ru-Ya Zhang, Chu-Fan Ma

**Affiliations:** 1 Department of Stomatology, Air Force Medical Center, The Fourth Military Medical University, Beijing 100142, China; rctian@cmu.edu.cn; 2 Graduate School, China Medical University, Shenyang 110002, China; 3 Department of Emergency and Oral Medicine, School and Hospital of Stomatology, China Medical University, Liaoning Provincial Key Laboratory of Oral Diseases, Shenyang 110002, China; 20202316@cmu.edu.cn

**Keywords:** bone marrow mesenchymal stromal cells, cellular senescence, rejuvenation, senile osteoporosis, aging

## Abstract

Bone marrow mesenchymal stromal cells (BM-MSCs) are multipotent cells present in bone marrow; they play a crucial role in the process of bone formation. Cellular senescence is defined as a stable state of cell cycle arrest that impairs the functioning of cells. Research has shown that aging triggers a state of senescence in BM-MSCs, leading to a reduced capacity for osteogenic differentiation and the accumulation of senescent cells, which can accelerate the onset of various diseases. Therefore, it is essential to explore mechanisms and strategies for the rejuvenation of senescent BM-MSCs. Senile osteoporosis (SOP) is a metabolic bone disease characterized by reduced bone formation. The senescence of BM-MSCs is considered one of the most important factors in the occurrence and development of SOP. Therefore, the rejuvenation of BM-MSCs for the treatment of SOP represents a promising strategy. This work provides a summary of the functional alterations observed in senescent BM-MSCs and a systematic review of the mechanisms that facilitate the rejuvenation of senescent BM-MSCs. Additionally, we analyze the progress in and the limitations associated with the application of rejuvenated senescent BM-MSCs to treat SOP, with the aim of providing new insights for the prevention and treatment of SOP.

## 1. Introduction

In 2006, the International Society for Cellular Therapy (ISCT) identified bone marrow mesenchymal stromal cells (BM-MSCs) as cells that demonstrate the ability to differentiate in vitro into osteoblasts, chondrocytes, adipocytes, neuronal cells, and myocytes, as well as exhibiting notable secretory, immunomodulatory, and homing properties [1]. Moreover, BM-MSCs offer structural assistance to hematopoietic stem cells (HSCs) within the bone marrow and secrete an array of growth factors (such as IL-11, LIF, M-CSF, and SCF) to facilitate hematopoiesis. Thus, BM-MSCs are crucial for bone formation and regeneration.

Cellular senescence is a stable state of cell cycle arrest accompanied by changes in biological function; it occurs in proliferating cells subjected to different types of stress [2,3,4]. Senescent cells can emerge at various stages of the lifespan, and their persistent presence and accumulation serve as indicators of aging. Senescent cells not only lose their normal cellular functions but also secrete a variety of pro-inflammatory cytokines, chemokines, proteases, and growth factors; this is collectively termed the senescence-associated secretory phenotype (SASP) [5,6,7]. Senescent cells frequently exert their effects through potent autocrine and paracrine activity, disrupting the functions of neighboring cells, compromising tissue homeostasis, and hindering tissue regeneration, thereby accelerating the aging process [6,8]. Research indicates that, with advancing age, the expression of aging-related genes in BM-MSCs increases, resulting in decreased proliferation, differentiation, and osteogenesis, while the adipogenic potential increases [9,10,11]. These changes lead to impaired bone formation.

Osteoporosis caused by aging (senile osteoporosis, SOP) is the most common age-related bone disease, characterized by a reduction in bone mass and the accumulation of adipocytes within the bone marrow cavity. BM-MSCs are multipotent cells present in the bone marrow; the functional decline caused by their senescence plays a crucial role in the occurrence and progression of SOP [12,13]. Therefore, the rejuvenation of senescent BM-MSCs represents a novel approach in the treatment of SOP, as well as in the field of regenerative medicine as a whole [9]. Recent studies on the rejuvenation of senescent BM-MSCs primarily focus on mitochondrial function, oxidative stress, and chronic inflammation. Other potential influencing factors include nutrient-sensing signaling pathways, autophagy, epigenetic regulation, and intercellular communication, as well as protein and lipid homeostasis. These intricate mechanisms interact and collaborate, working synergistically. This work provides a summary of the functional alterations observed in senescent BM-MSCs and a systematic review of recent research advancements regarding the mechanisms that facilitate the rejuvenation of senescent BM-MSCs. In addition, we analyze the progress in and the limitations associated with the application of rejuvenated senescent BM-MSCs to treat SOP, with the aim of offering new research directions for the prevention and treatment of SOP.

## 2. Functional Changes in BM-MSC Senescence

**Figure 1:**
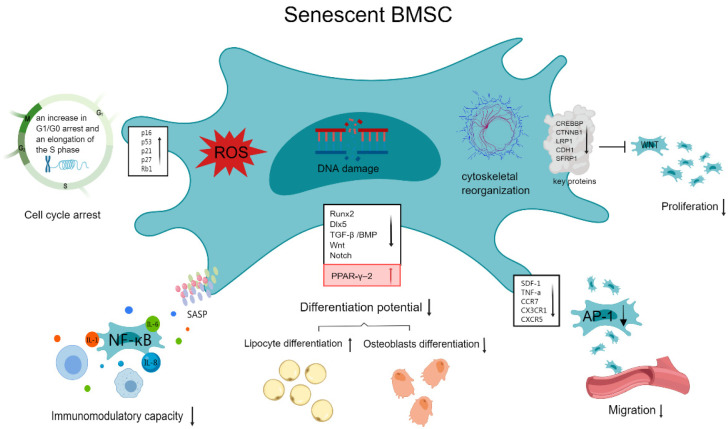
Functional changes in senescence BM-MSCs, including cell cycle arrest; decreased proliferation, differentiation, immune regulation, paracrine, migration, and homing abilities; and, simultaneously, the balance of differentiation shifts towards adipocytes.

The main characteristics of senescent BM-MSCs include changes in cell morphology, decreased proliferative capacity, weakened migratory homing ability, altered differentiation potential, and changes in paracrine secretion (Figure 1).

### 2.1. Cell Morphology

Changes in cell morphology are one of the hallmarks of cellular senescence. In contrast to the uniform fibroblast-like spindle shape of young BM-MSCs, senescent BM-MSCs exhibit a flattened and enlarged appearance, along with abnormal nuclear morphology, and a loss of heterochromatin stability and nuclear lamina integrity [14]. Additionally, senescent BM-MSCs also exhibit features such as a granular cytoplasm, a significant increase in the cytoplasmic area, a reduction in the nuclear–cytoplasmic ratio, and difficulty in digestion with trypsin [15]. Furthermore, extensive research has shown a close link between the morphological characteristics of senescent BM-MSCs and their biological functions, with some scholars finding that morphological features can highly predict the osteogenic capacity of BM-MSCs [16].

### 2.2. Cell Proliferation and Self-Renewal

Leonard Hayflick identified that normal human cells cultivated in vitro possess a finite potential for division before undergoing senescence. Typically, these cells undergo about 50 divisions before entering a state of senescence, leading to the recognition of the “Hayflick limit” phenomenon [17]. Prolonged interruption of the cell cycle is triggered by elevated levels of molecular markers such as p53, p21, p27, p16, and Rb1 [18]. Studies have shown that the mitotic activity of BM-MSCs from elderly donors is nearly 1.5 times lower than that of younger donors, with their proliferation rate being only about half that of younger donors [19]. Furthermore, there has been an observed increase in G1/G0 arrest and the elongation of the S phase in BM-MSCs obtained from elderly donors [10]. Research has indicated that several key proteins (CREBBP, CTNNB1, LRP1, CDH1, and SFRP1) are able to regulate the Wnt signaling pathway, thereby affecting the proliferation of senescent BM-MSCs [20].

### 2.3. Cell Migration and Homing

The number of BM-MSCs in the bone marrow decreases as age advances, and their migratory capacity also decreases. Studies have confirmed that the turnover rate of actin in senescent BM-MSCs is reduced, with a decrease in the dynamic actin cytoskeleton, leading to a diminished response to biological and mechanical signals, which, in turn, results in a reduced level of cell migration [21]. Furthermore, the inhibition of the AP-1 pathway is also associated with the decreased migratory capacity of senescent BM-MSCs [22]. Additionally, research has shown that the expression of some cytokines and chemokine receptors, such as stromal cell-derived factor 1 (SDF-1), tumor necrosis factor-alpha (TNF-α), and chemokine receptors CCR7, CX3CR1, and CXCR5, is reduced in senescent BM-MSCs [23], thereby disrupting the potential of BM-MSCs to be activated and mobilized to the site of injury.

### 2.4. Cell Differentiation

The expression of the osteoblast-specific transcription factors Runx2 and Dlx5, as well as osteoblast marker collagens and Osteocalcin, is decreased in elderly BM-MSCs. Conversely, the adipogenic potential of elderly BM-MSCs is increased [24]. Extensive research has confirmed that the Notch signaling pathway, the TGF-β/BMP signaling pathway, and the Wnt signaling pathway, which promote osteogenic differentiation, are inhibited in senescent BM-MSCs [25]. Furthermore, the expression of the peroxisome proliferator-activated receptor γ-2 (PPAR-γ–2) signaling pathway, which promotes adipogenesis, is elevated in elderly BM-MSCs [26,27]. This is the most characteristic functional change in senescent BM-MSCs.

### 2.5. Paracrine and Senescence-Associated Secretory Phenotype and Immune Regulation

Senescent cells produce a range of pro-inflammatory cytokines, chemokines, proteases, and growth factors through paracrine, collectively known as the SASP, which can induce an inflammatory state and senescence in surrounding normal cells [7]. There is a mutually reinforcing relationship between senescence and inflammation [6]. Recent studies indicate that the secretion of SASP by senescent BM-MSCs causes inflammation in the bone marrow microenvironment, and the exacerbated inflammatory response can promote the adipogenic differentiation of bone marrow mesenchymal stem cells, leading to bone loss [28]. Additionally, it has been reported that BM-MSCs can regulate both innate and adaptive immune responses [29]. However, the immune regulatory activity of senescent BM-MSCs was reduced [30]. The immune regulatory function of senescent BM-MSCs is impaired, as evidenced by their reduced ability to suppress the M1 polarization of macrophages [31], which further exacerbates the inflammatory response.

## 3. Mechanisms of Rejuvenation in Senescent BM-MSCs

Mitochondrial function, oxidative stress, and chronic inflammation are currently the focal points of research on the mechanisms of rejuvenation in senescent BM-MSCs. Other potential influencing factors include nutrient-sensing signaling pathways, autophagy, epigenetic regulation, and intercellular communication, as well as protein and lipid homeostasis. These mechanisms are not isolated; rather, they are interconnected through complex networks.

### 3.1. The Close Relationship Between Mitochondria, Oxidative Stress, and Chronic Inflammation

#### 3.1.1. Mitochondrial Function

**Figure 2:**
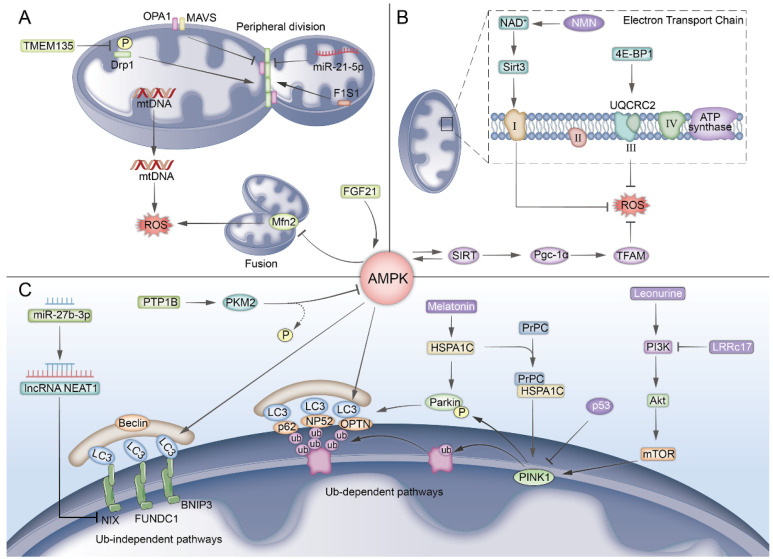
Inhibition of the production of mitochondrial ROS to promote the rejuvenation of senescent BM-MSCs (**A**–**C**).

Mitochondria, known as “cellular powerhouses”, are involved in the regulation of various cellular functions [32]. Mitochondrial function declines with age, which is the primary reason for the production of ROS during the aging process [33,34]. When mitochondrial function is impaired, the incomplete reduction of molecular oxygen during the OXPHOS process becomes more significant, leading to the production of ROS [35]. Increased ROS can cause mutations in mitochondrial DNA (mtDNA), which in turn impairs OXPHOS, leading to a vicious cycle of mitochondrial homeostasis collapse [36]. Recently, “antiaging mitochondrial therapy” has garnered increased attention [37] (Figure 2).

• Mitochondrial dynamics

The continuous process of mitochondrial fusion, fission, movement, and crista remodeling under the action of related proteins is termed “mitochondrial dynamics”. Disruptions in mitochondrial dynamics can lead to mitochondrial dysfunction and the induction of ROS [38,39,40]. An increase in the level of the mitochondrial fusion protein Mfn2 promotes mitochondrial fusion [41], which in turn has a negative impact on bone formation [42]. The exogenous overexpression of fibroblast growth factor 21 (FGF21) can reduce the level of Mfn2 through the AMPK signaling pathway, inhibit the production of ROS, and effectively rejuvenate senescent BM-MSCs [41].

Mitochondrial fission can be divided into two types based on spatial differences: midzone division and peripheral division. Among these, midzone division is a marker of healthy mitochondria. However, as cells undergo senescence, the phosphorylation and expression levels of dynamin-related protein 1 (Drp1), the main regulatory factor of peripheral division, increase [43]. This results in a significant increase, while the smaller products of this peripheral fission often lack replicable DNA—an indicator of unhealthy mitochondria [44]. Research indicates that defects in transmembrane protein 135 (TMEM135) can promote the phosphorylation of Drp1 at serine 637 in BM-MSCs [45], impair mitochondrial fission, and disrupt the critical mitochondrial energy metabolism during osteogenesis. However, thus far, research targeting TMEM135 has only been conducted in young BM-MSCs. Further investigation is required to determine whether the regulation of TMEM135 can effectively rejuvenate senescent BM-MSCs. The mitochondrial antiviral signaling protein MAVS is a critical membrane protein that functions as a scaffold to stabilize the guanosine triphosphatase OPA1 on the mitochondrial membrane and interacts with it to uphold mitochondrial dynamic homeostasis [46]. The inactivation of the *MAVS* and *OPA1* genes compromises the mitochondrial structure and integrity. Research indicates that upregulating the expression of the *MAVS* and *OPA1* genes can effectively promote the rejuvenation of senescent BM-MSCs [14]. Furthermore, epigenetic regulation is essential for mitochondrial dynamics. For example, the miR-21-5p levels decrease in senescent BM-MSCs. Upregulating miR-21-5p enhances mitochondrial fission and promotes the recovery of stemness in senescent BM-MSCs [47]. Mitofilin is a mitochondrial inner membrane protein. There is evidence that, in the absence of mitofilin, the mitochondrial membrane potential of senescent BM-MSCs is impaired, resulting in the formation of swollen and inefficient mitochondria. Thus, the upregulation of mitofilin represents a potential strategy for the rejuvenation of BM-MSCs [48].

• Mitophagy

Recent work suggests that the regulation of mitophagy can help to restore the function of senescent BM-MSCs [49]. Mitochondrial autophagy typically occurs through the ubiquitin-mediated PINK1/Parkin pathway. PINK1 is a serine/threonine kinase located on depolarized mitochondria, while Parkin is an E3 ubiquitin ligase that catalyzes the transfer of ubiquitin to mitochondrial substrates. Compared to normal cells, an increase in P53 in senescent BM-MSCs inhibits the mitochondrial translocation of Parkin and the activation of Parkin’s E3 ubiquitin ligase [50], leading to the inhibition of mitophagy. Research has shown that inhibiting P53 and/or upregulating the expression of Parkin helps to rejuvenate senescent BM-MSCs [50,51]. Heat shock protein 1L (HSPA1L) is a molecular chaperone that can bind to PINK1 and Parkin, participating in mitochondrial autophagy [52]. Upregulating HSPA1L can significantly increase the levels of Parkin and PINK1 in the mitochondria of senescent BM-MSCs, making it an effective target for BM-MSC rejuvenation [53]. Another pathway—non-ubiquitin-dependent mitophagy—is dominated by mitophagy receptors. Mitophagy receptors such as BNIP3/NIX (also known as BNIP3L) and FUNDC1 contain a conserved LC3 interaction region (LIR), which allows them to directly bind to autophagy-related proteins (ATGs, such as LC3) via the LIR motif, thereby initiating autophagy [54]. In addition, the highly expressed long non-coding RNA NEAT1 in senescent BM-MSCs can regulate the expression of BNIP3L through the sponging of miR-27b-3p, resulting in imbalanced mitophagy. Research shows that si-NEAT1 contributes to the rejuvenation of senescent BM-MSCs [55].

AMPK also plays an important role in the process of mitophagy [56]. QianKun Yang et al. [57] found that, in senescent BM-MSCs, PTP1B could inactivate AMPK through dephosphorylation mediated by PKM2. After the knockdown of PTP1B, the AMPK signaling pathway was significantly activated, promoting the upregulation of the expression of autophagy-related genes *LC3* and *Beclin1*, thereby alleviating the senescence of BM-MSCs. Furthermore, mitophagy is regulated by the PI3K/Akt/mTOR pathway. The upregulation of LRRc17 in senescent BM-MSCs leads to the suppression of the PI3K/Akt/mTOR pathway [58]. Leonurine activates the PI3K/Akt/mTOR pathway, which results in the upregulation of PINK1 and Parkin expression, promotes mitophagy, reduces the ROS levels in senescent BM-MSCs, and rejuvenates BM-MSCs under senescence [59].

• Mitochondrial energy metabolism

Key regulatory factors of mitochondrial metabolism, including Pgc-1α, AMPK, SIRT, and mitochondrial transcription factor A (TFAM), are suppressed with increasing age. The upregulation of these factors can effectively prevent the excessive production of ROS and promote the rejuvenation of senescent BM-MSCs [48,60]. Oxidative phosphorylation (OXPHOS) and the mitochondrial respiratory chain (MRC) play a central role in mitochondrial energy metabolism, determining the fates of BM-MSCs [61]. Huan Wang et al. [62] discovered that the exogenous supplementation of NMN, a precursor of NAD+, could reduce the loss of MRC complex I in BM-MSCs and decrease the ROS levels through its effects on NAD+ and Sirt, thereby alleviating the senescence of BM-MSCs. Additionally, the eukaryotic translation initiation factor 4E-binding protein 1 (4E-BP1) can upregulate the expression of the core component UQCRC2 in the mitochondrial OXPHOS complex III and prevent the excessive production of ROS, thereby rejuvenating senescent BM-MSCs [63].

Currently, the majority of mitochondrial targeting relies on attraction towards the highly negative inner mitochondrial membrane (IMM), mediated by positive charges [64]. However, an excessive number of positive charges can depolarize the mitochondrial membrane [65], leading to the release of cytochrome c and the activation of the apoptotic pathway. Additionally, excessive positive charges may result in increased cytotoxicity due to the more severe disruption of the plasma membrane [66]. Therefore, when the mitochondrial membrane potential decreases due to BM-MSCs’ senescence, the accuracy and safety of existing mitochondrial-targeting moieties may be affected; this needs further clarification.

- Mitochondrial dynamics: inhibiting mitochondrial fusion and asymmetric peripheral fission can suppress mitochondrial ROS production.
- Mitochondrial energy metabolism: regulating the activity of the OXPHOS complex can inhibit mitochondrial ROS production.
- Mitochondrial autophagy: Promoting ubiquitin-dependent (PINK1-Parkin) and ubiquitin-independent mitochondrial autophagy pathways can inhibit mitochondrial ROS production.

#### 3.1.2. Oxidative Stress

Mitochondrial dysfunction is a primary contributor to excessive ROS production. However, organisms also possess the ability to simultaneously inhibit ROS. Antioxidants, such as superoxide dismutase (SOD), catalase (CAT), glutathione peroxidase (GPX), glutathione reductase (GR), NAD(P)H quinone oxidoreductase 1 (NQO1), and heme oxygenase-1 (HO-1), play a role in the removal of ROS.

Activating the AMPK-Sirt1 signaling pathway and enhancing the deacetylation of the downstream antioxidant stress target FOXO3a can promote the expression of antioxidant enzyme genes, especially *SOD1* and *SOD2*, and inhibit the senescence of BM-MSCs [60,67,68]. Further research has demonstrated that the overexpression of Sirt3 in BM-MSCs can augment their antioxidant capacity through the upregulation of both the expression and activity of SOD2 [69]. In addition, the Erk1/2 signaling pathway can increase the phosphorylation of p90RSK and CREB; enhance the activity of SOD, CAT, and GPX; and significantly reduce the ROS levels [70].

Additionally, the transcription factor nuclear factor erythroid 2-related factor 2 (NRF2) is a primary regulator of cellular redox homeostasis [71]. It participates in the initiation of antioxidant enzyme expression and induces the transcription of antioxidant genes under oxidative stress [72]. Due to the relatively low efficiency of systemic supplementation with exogenous antioxidants in clearing ROS, it is challenging to restore the vitality of senescent BM-MSCs. Therefore, promoting the NRF2 pathway, which facilitates the production of endogenous antioxidants in the body, may be an effective means to enhance the antioxidant capacity of senescent BM-MSCs. Studies have shown that 1,25-Dihydroxyvitamin D3 (1,25(OH)2D3) enhances NRF2 transcription through VDR mediation, thereby inhibiting oxidative stress and the production of SASP []. Another study has shown that [73] a second-generation active vitamin D analog, Eldecalcitol (ED-71), can regulate the SIRT1-NRF2 signaling pathway, enhance its antioxidant capacity, inhibit the levels of ROS in BM-MSCs, and effectively rejuvenate senescent BM-MSCs.

#### 3.1.3. Chronic Inflammation

The concept of “inflammaging” was first introduced in 2000 by Franceschi C et al.; it refers to the higher levels of inflammatory markers often found in the cells and tissues of older organisms [74]. The fundamental characteristic of inflammatory aging is the production of SASP. SASP has the potential to facilitate chronic inflammation and trigger the process of normal cellular senescence. Accumulated senescent cells secrete high levels of pro-inflammatory factors, including IL-1β, IL-6, IL-8, IL11, and tumor necrosis factor-α (TNF-α), which further promote the senescence of surrounding cells in a paracrine manner [75]. Recent studies have confirmed that the cGAS-STING pathway is a key signaling pathway that promotes the production of SASP [76]. Suppressing the expression of inflammatory factors helps to restore the homeostasis of the bone marrow microenvironment, which is an effective strategy for the rejuvenation of senescent BM-MSCs [77].

A large number of studies have confirmed the close relationship between oxidative stress, inflammation, and aging [78]. Therefore, the oxidation–inflammatory theory of aging has been proposed, also known as the oxi-inflammaging theory [79]. NF-κB is the key link between inflammation and oxidative stress [80]. Oxidative stress can activate NF-κB, promoting the release of inflammatory factors, which further exacerbate oxidative stress, creating a vicious cycle that leads to increased cellular senescence. Research has confirmed that [81] both an increase in PGC-1α and a decrease in NAP1L2 can inhibit the NF-κB signaling pathway, promoting the rejuvenation of senescent BM-MSCs [82,83]. In addition, anti-inflammatory and antioxidant effects often play a synergistic role in the antiaging process. For instance, the natural antioxidant lutein can significantly increase the expression of SOD and reduce the levels of inflammatory factors TNFα, IL-1β, and IL-6, thereby decreasing the expression of senescence-associated markers in BM-MSCs [84].

Although anti-inflammatory pathways have shown some potential for the rejuvenation of senescent BM-MSCs, their application faces numerous limitations and challenges. The inflammatory response is a complex process, and anti-inflammatory pathways may interfere with the normal immune response in the bone marrow microenvironment, leading to a decline in immune function. In addition, the immune system possesses an extraordinary ability to remember and respond to different stimuli and experiences, leading to heterogeneity in immune aging among individuals. This heterogeneity may be attributed to differences in the types, doses, intensities, and temporal sequences of the antigenic stimuli to which each person is exposed. Therefore, a more comprehensive approach that takes various factors into consideration may be necessary.

### 3.2. Nutrient-Sensing Network

**Figure 3:**
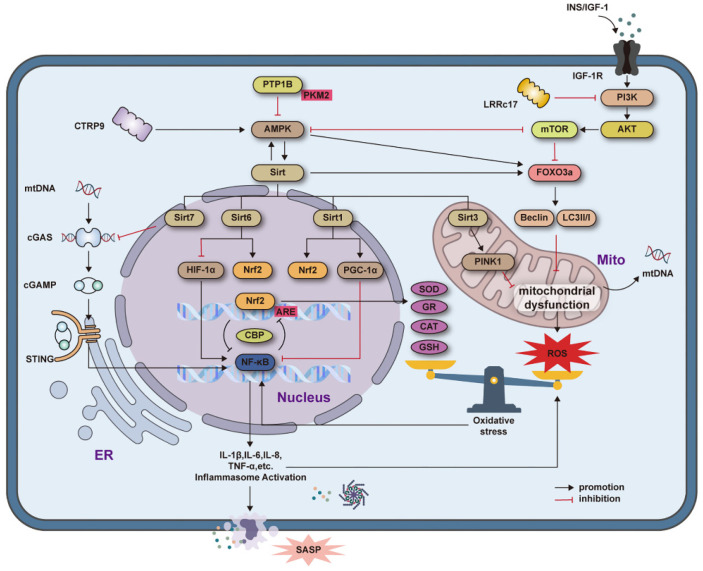
The interaction between nutrient-sensing pathways and mitochondrial oxidative stress and chronic inflammation. The interplay among IIS, AMPK, mTOR, and Sirt involves regulating the Nrf2 and NF-κB signaling pathways and mitochondrial function through cascading interactions. Dysfunction in the mitochondria releases mtDNA, triggering inflammatory responses through the cGAS-STING signaling pathway. The release of inflammatory markers and mitochondrial dysfunction further induce oxidative stress, thereby exacerbating chronic inflammation and mitochondrial damage. By modulating key components in the described signaling pathways, stimuli can facilitate the rejuvenation of senescent BM-MSCs.

The nutrient-sensing network is a key regulator of cellular activity. It not only influences energy metabolism during aging but also plays a crucial role in regulating mitochondrial function, oxidative stress, and autophagy. This network primarily consists of four key pathways: the insulin/insulin-like growth factor 1 (IGF-1) signaling pathway (IIS), the sirtuins/NAD+ pathway, the AMPK pathway, and the mTOR pathway (Figure 3).

#### 3.2.1. IIS

The IIS pathway is one of the most evolutionarily conserved mechanisms of aging control, with its apex being growth hormone (GH) produced by the pituitary gland, which stimulates the secretion of insulin/IGF-1 by acting on GH receptors in liver cells. Additionally, IIS has multiple downstream targets. Intracellular signaling cascades involve the PI3K-AKT and mTORC1 nutrient signaling networks, forming the GH/IGF-1/PI3K/AKT/mTORC1 axis. This is the first growth cascade axis historically discovered to be associated with the control of senescence. Dietary restriction can delay aging by inhibiting this axis [85,86]. However, a growing number of studies have shown that impaired IGF-1 signaling during the aging process can reduce the osteogenic capacity and migratory ability of senescent BM-MSCs [87,88]. The above evidence indicates that while inhibiting IIS may delay cellular senescence, the reduced levels of IGF-1 in the bone matrix during aging may not be adequate for new bone formation. Thus, it is hypothesized that maintaining optimal levels of IGF-1 could be an important strategy for rejuvenating the osteogenic vitality of senescent BM-MSCs.

#### 3.2.2. mTOR Signaling Pathway

MTOR was first identified in research on rapamycin, comprising two distinct complexes, mTORC1 and mTORC2 [89]. It can respond to nutrients (including glucose and amino acids) as well as stressors (such as hypoxia and low energy), regulating processes such as cell growth, proliferation, metabolism, and protein synthesis. The mTOR signaling pathway is widely recognized as a crucial regulator in the senescence process of BM-MSCs [90,91]. The negative regulatory effect of mTOR on PGC-1α promotes the accumulation of intracellular ROS, induces an oxidative stress state, and accelerates the senescence process of BM-MSCs. Additionally, mTOR enhances the signaling of the key inflammatory regulator NF-κB by promoting the phosphorylation and nuclear translocation of p65, thereby negatively influencing the osteogenic differentiation of BM-MSCs. Research by Guoxiang Liu and colleagues has found that C-PC can delay the senescence of BM-MSCs in vivo by inhibiting the PI3K/AKT/mTOR signaling pathway [92]. Thus, inhibiting mTOR signaling may be a promising therapeutic target for rejuvenating senescent BM-MSCs.

#### 3.2.3. Sirtuins/NAD+ Pathway

Sirtuins, a family of histone deacetylases, are widely regarded as longevity genes. They play a vital role in DNA repair, protein homeostasis, cellular metabolism, inflammatory regulation, and circadian rhythm control [93], contributing significantly to the prevention of BM-MSC senescence [94]. Among them, SIRT1, SIRT3, SIRT6, and SIRT7 have been extensively studied for their antiaging properties, and their overexpression may serve as an effective strategy for rejuvenating senescent BM-MSCs [95].

The overexpression of SIRT1 can further promote the proliferation and osteogenesis of senescent BM-MSCs by enhancing the deacetylation and nuclear translocation of Bmi1 [96]. SIRT1 is also an important antioxidant [97]. The deacetylation of the key antioxidant response transcription factor FOXO3a by SIRT1 can restore the effects of oxidative stress on the osteogenic potential of senescent BM-MSCs [98]. Another study has indicated that mechanical stretching can regulate the antioxidant defense capabilities of BM-MSCs by activating SIRT1 [99]. Moreover, SIRT1 also can inhibit BM-MSCs senescence by enhancing TPP1 expression and increasing telomerase activity [100].

SIRT3, containing an N-terminal mitochondrial-targeting sequence, is predominantly localized within mitochondria. SIRT3 is decreased in senescent BM-MSCs [95]. The overexpression of Sirt3 can reduce the accumulation of advanced glycation end products (AGEs) in mitochondria and significantly alleviate the senescence phenotype of BM-MSCs [13]. Fei Liu et al. have confirmed that the S-nitrosation of cysteine residues can enhance SIRT3 activity by forming persulfides, thereby stabilizing heterochromatin and mitochondrial homeostasis to combat the senescence of BM-MSCs [101].

SIRT6 catalyzes deacetylation and mono-ADP-ribosylation reactions, playing a crucial role in cellular senescence [102]. SIRT6 protects BM-MSCs from oxidative stress by binding directly to the NRF2 promoter region and exerting antioxidant effects through the NRF2-HO-1 pathway [103]. Additionally, SIRT6 regulates glucose metabolism by maintaining the deacetylation of H3K9 at the promoters of glycolytic genes and inhibiting HIF1α [104]. Thus, the overexpression of SIRT6 is a novel mechanism to rejuvenate senescence-related functional decline in BM-MSCs.

Recent studies have confirmed that a deficiency of SIRT7 can lead to genomic instability, metabolic dysfunction, and accelerated senescence of BM-MSCs [105,106]. SIRT7 forms a complex with nuclear lamina proteins and heterochromatin proteins, protecting chromatin’s structure through the cGAS-STING pathway, maintaining the repressive state of heterochromatin at the nuclear periphery, and controlling innate immune regulation [107]. Therefore, upregulating SIRT7 can rejuvenate the senescent process of human BM-MSCs (Table 1).

The expression of NAD+ declines with increasing age, and the expression and activity of nicotinamide phosphoribosyltransferase (NAMPT) and sirtuins also significantly decrease [108]. Due to the barrier of the cell membrane, NAD+ cannot easily enter the human body. Therefore, the exogenous supplementation of its direct precursor NMN is the best way to increase NAD+ levels in the body [109]. NMN can rejuvenate the senescence of BM-MSCs through the upregulation of SIRT1 and SIRT3 expression, as well as by improving mitochondrial function [62,110].

**Table 1:** Upstream and downstream targets and mechanisms of SIRT.

Upstream Stimulation	Sirtuin	Downstream Targets	Mechanism	References
Mechanical stretching; NMN; NAMPT	SIRT1	Bmi1	Enhance the deacetylation and nuclear translocation of Bmi1	[96]
		FOXO3a	Enhance antioxidant capacity	[98]
		TPP1	Increase telomerase activity	[100]
S-nitrosation of cysteine; NMN	SIRT3	mitochondria	Reduce the accumulation of AGEs	[13]
		cell nucleus	Stabilize heterochromatin	[111]
	SIRT6	NRF2	Exert antioxidant effects through the NRF2-HO-1 pathway	[103]
		H3K9; HIF1α	Regulate glucose metabolism	[104]
	SIRT7	cGAS-STING	Protect chromatin structure; control innate immune regulation	[107]

#### 3.2.4. AMPK Pathway

The responsiveness of the AMPK signaling pathway declines with age [112]. Extensive research has shown that AMPK activation can phosphorylate key proteins across multiple signaling pathways, restore energy balance, and slow the senescence of BM-MSCs [113]. The exogenously supplemented macrophage migration inhibitory factor (MIF) can activate the AMPK-FOXO3a signaling pathway by interacting with CD74, thereby enhancing the proliferative rate of senescent BM-MSCs and mediating paracrine signaling capabilities [114]. Qun Li et al. have shown that C1q and tumor necrosis factor-related protein 9 (CTRP9) can activate the PGC-1α/AMPK signaling pathway, decrease cellular oxidative stress, and present a novel therapeutic strategy to rejuvenate the senescence of BM-MSCs [115]. AMPK also reciprocally inhibits with mTOR [116]. Under conditions of sufficient nutrition, AMPK is inactive, while mTOR is active. Under conditions of energy deficiency, the increased activity of AMPK leads to a decrease in mTOR activity. Low levels of mTOR can further lead to a slowdown in cell growth and a reduction in protein synthesis. AMPK can also directly regulate protein synthesis by phosphorylating the negative regulatory factor of the elongation factor eEF2K [117]. Finally, AMPK and sirtuins/NAD+ are important intracellular sensors of nutrient deficiency. AMPK can activate SIRT1, integrating two low-energy sensing systems into a unified response as part of a positive feedback loop [118], and synergistically participate in the differentiation of BM-MSCs [119]. Studies have shown that Kartogenin (KGN) can improve the antioxidant characteristics of senescent BM-MSCs in a dose-dependent manner by activating the AMPK-SIRT1 signaling pathway [120].

These findings suggest that inhibiting the IIS and mTORC1 pathways and/or upregulating AMPK and sirtuins can promote the rejuvenation of senescent BM-MSCs. However, IIS and mTOR play essential roles in cell proliferation during tissue repair, and excessive inhibition may lead to complications such as insulin resistance and impaired tissue regeneration [121]. Moreover, the overactivation of AMPK has been linked to tau protein hyperphosphorylation, which can cause neuronal damage and increase the risk of Alzheimer’s disease. Therefore, the effectiveness of nutrient-sensing pathways in BM-MSC rejuvenation depends on the timing and extent of the intervention. Precise regulation is crucial to optimizing benefits while minimizing potential risks [122].

### 3.3. Autophagy

The dysregulation of autophagy is closely linked to cellular senescence [123,124]. This imbalance contributes to key molecular processes associated with aging, including disrupted proteostasis, mitochondrial dysfunction, oxidative stress, and epigenetic alterations, ultimately driving cellular senescence [50,125].

The central negative regulator of autophagy is the mechanistic target of the rapamycin (mTOR) pathway. Inhibiting mTOR promotes autophagy in senescent BM-MSCs and facilitates their osteogenic differentiation [126]. Inhibiting the activation of the PI3K/AKT/mTOR pathway mediated by zinc finger-containing Asp-His-His-Cys domain protein 5 (ZDHHC5) can increase the level of autophagy in senescent BM-MSCs, promoting rejuvenation [92]. Autophagy can also be directly regulated by the AMPK pathway. Apelin activates the AMPK signaling pathway and upregulates the expression of autophagy proteins Beclin and LC3II/I to rejuvenate senescent BM-MSCs [127]. OPTN is another autophagy receptor that plays a central role in selective autophagy. FABP3 is an OPTN substrate mainly expressed in bone tissue. The overexpression of OPTN or degradation of FABP3 can enhance the osteogenic potential of senescent BM-MSCs by activating autophagy [128]. In addition, downregulating the autophagy inhibitor protein P62 can enhance the expression of autophagy-related genes *LC3*,* ULK1*, *Atg7*, and *Atg12*, thereby promoting the rejuvenation of senescent BM-MSCs [129].

In fact, autophagy plays a dual role in the senescence of BM-MSCs. While it supports cellular homeostasis, excessive autophagy can exacerbate DNA damage and promote protein aggregation. This paradox underscores the need for a deeper understanding of autophagy’s mechanisms under different conditions to develop more precise and effective strategies for BM-MSC rejuvenation.

### 3.4. Intercellular Communication

Multiple studies have shown that exposure to young serum can restore youthful characteristics in senescent cells [130,131,132]. This suggests that regulating intercellular communication may be a promising strategy for rejuvenating senescent BM-MSCs. Extracellular vesicles (EVs) are considered important carriers of intercellular signaling. Research indicates that inhibiting EVs carrying long-chain C24:1 ceramide can rejuvenate the senescent BM-MSCs [133]. In addition, Yan Zhang et al.’s research shows that the autonomic nervous system (ANS) regulator γ-oryzanol promotes the proliferation and osteogenic differentiation of senescent BM-MSCs by reducing the production of paracrine neuropeptide Y (NPY) [134]. Other research has indicated that CC chemokine ligand 3 (CCL3) accumulates in the serum of naturally aged mice, accompanied by bone aging phenotypes and an imbalance in BM-MSC differentiation. Blocking the expression of CCL3 in vivo with neutralizing antibodies can improve the osteogenic differentiation potential of BM-MSCs in aged mice [135].

However, the bone marrow microenvironment where BM-MSCs reside changes constantly with aging. Collagen and elastin in the extracellular matrix undergo cross-linking and denaturation, worsening physical support and intercellular signaling. This may increase the difficulty of rejuvenating BM-MSCs through intercellular communication.

### 3.5. Others

The accumulation of misfolded or damaged proteins, as well as the disruption of the “Protein Homeostasis Network”, can adversely affect proteostasis, which is one of the significant causes of senescence [136]. Melatonin can maintain mitochondrial proteostasis through the mitochondrial unfolded protein response (UPRmt) regulator PDI-6, restoring the function of senescent BM-MSCs [137]. The expression of Zmpste24 decreases with age, resulting in the accumulation of Pre-LaminA in the nucleus, while the LaminA content that maintains and stabilizes the nucleus decreases. Ruici Yang et al. have shown that physical exercise can reverse the accumulation of Pre-LaminA caused by the deficiency of Zmpste24 in aged mouse BM-MSCs, improving the biological activity of senescent BM-MSCs [138]. In recent years, an increasing number of scholars have found that ribosomes are closely related to proteostasis and senescence. Kevin C. Stein and colleagues reported that Arg, Lys, and Pro residues are closely related to the increased pausing of ribosomes during the senescence process [139]. The exacerbation of ribosomal pausing at specific sites can lead to increased ribosomal collisions, causing an overload of ribosome-associated quality control (RQC) and the aggregation of nascent polypeptides, thereby disrupting proteostasis. However, whether the regulation of ribosomes can rejuvenate functional changes related to the senescence of BM-MSCs still requires further research.

Lipidomics, as a burgeoning branch of metabolomics, has been demonstrated in recent years to be closely related to cellular senescence [140]. Apolipoprotein E (APOE) may mediate the senescence of human BM-MSCs by regulating the distribution of lipids on the nuclear envelope or in a lipid-binding-dependent manner. The knockout of APOE can rejuvenate senescent cells [141].

Additionally, a growing body of research suggests that epigenetic changes are key drivers of mammalian aging [142]. Previous studies have comprehensively summarized the epigenetic targets and pathways involved in the rejuvenation of senescent BM-MSCs [142,143,144]. However, while epigenetic regulation can partially reverse the accumulation of abnormal gene modifications, it cannot correct underlying genetic defects. Therefore, epigenetic regulation alone may be insufficient and should be combined with other regulatory mechanisms for optimal efficacy.

## 4. Rejuvenating Senescent BM-MSCs to Treat SOP

Over the past few decades, research on the pathogenesis of osteoporosis has shifted from an “estrogen-centric” perspective to a “cellular-senescence-centric” perspective [145]. Recent studies indicate that SOP can be characterized as a stem cell disorder, and the senescence of BM-MSCs is considered one of the most important factors in the occurrence and development of SOP [4].

### 4.1. The Senescence of BM-MSCs Leads to SOP

**Figure 4:**
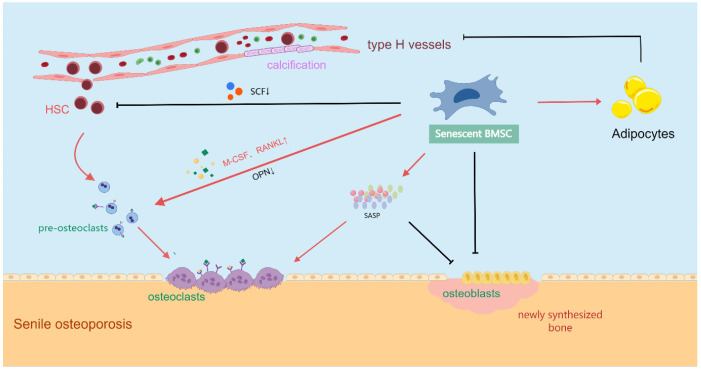
The influence of senescent BM-MSCs on the bone marrow microenvironment.

Senescent BM-MSCs impact the bone formation process by affecting the bone marrow microenvironment, ultimately leading to SOP (Figure 4). First, the senescence of BM-MSCs leads to increased differentiation towards adipocytes while reducing osteogenic differentiation [146]. An increase in bone marrow adipocytes can inhibit the formation of H-type blood vessels through growth factors such as leptin and adiponectin, thereby impairing bone formation [147]. Second, as individuals age, the paracrine function of BM-MSCs changes. The levels of M-CSF, RANKL, and IL-6 increase, while the OPN levels decrease, promoting the differentiation of pre-osteoclasts into osteoclasts [148,149]. The decreased secretion of growth factors such as SCF inhibits the self-renewal and differentiation of hematopoietic stem cells. Other studies have shown that EVs derived from senescent BM-MSCs can infiltrate the vascular tissue, promoting the osteogenic transdifferentiation of vascular smooth muscle cells, aggravating vascular calcification, and inhibiting bone formation [150]. In addition, the SASP secreted by senescent BM-MSCs causes the bone marrow microenvironment to enter a state of chronic inflammation, impairing the functioning of adjacent cells. The increase in SASP not only affects the bone formation activity of osteoblasts but also enhances bone resorption by osteoclasts [142]. Finally, the senescence of BM-MSCs leads to the abnormal synthesis and secretion of components such as collagen, resulting in structural changes in the extracellular matrix (ECM); these subsequently affect cell-to-cell signaling in the bone marrow microenvironment [146,151]. Therefore, rejuvenating senescent BM-MSCs can promote bone formation under SOP conditions by improving the bone marrow microenvironment.

### 4.2. The Application of Rejuvenating Senescent BM-MSCs to Treat SOP

In the previous section, we summarized the mechanisms of BM-MSC rejuvenation at the molecular level. However, only some of these targets have been applied in preclinical studies on SOP, and further exploration is needed to determine whether the remaining mechanisms can be utilized to treat SOP.

#### 4.2.1. Drugs or Regulators

Several preclinical studies have indicated that drugs or regulators can improve the bone density of osteoporotic animals through the rejuvenation of senescent BM-MSCs. For instance, the exogenous supplementation of 1,25(OH)2D3 and alpha-ketoglutarate (αKG) could promote an increase in bone mineral density in naturally aged mice and mitigate SOP through regulating the enrichment of H3K9me3 and H3K27me3 in senescent BM-MSCs [152,153]. Dendrobium officinale polysaccharides (DOPs) and PQQ have been demonstrated to facilitate the activation of the NRF2 signaling pathway, which ameliorated SOP in aging mice [154,155]. CCG could promote bone formation in aging mice through upregulating the TAZ signaling pathway in senescent BM-MSCs [156]. Resveratrol (RESV) improved mitochondrial function in senescent BM-MSCs through upregulating mitofilin, thereby promoting osteogenesis in aging mice [48].

In addition, a key characteristic of an effective treatment measure is a diverse array of mechanisms [136]. For example, the local administration of tetramethylpyrazine can not only aid in suppressing the senescent characteristics of BM-MSCs through regulating the Ezh2-H3K27me3 pathway; it also boosts metabolic function and anti-inflammatory mechanisms to alleviate SOP through the modulation of the AMPK-mTOR-Hif1a-VEGF signaling pathway [157]. Moreover, on one hand, the external administration of melatonin suppresses the NF-κB signaling pathway and reduces the synthesis of inflammatory factors in senescent BM-MSCs; on the other hand, melatonin can induce the upregulation of NSD2, restoring the equilibrium between the H3K36me2 and H3K27me3 levels for the promoters of osteogenic genes in senescent BM-MSCs. This strategy ultimately led to the alleviation of osteoporosis in aging mice [158,159] (Table 2).

Senolytics are a class of drugs that can specifically eliminate senescent cells [12]. They achieve this by temporarily inducing the failure of the senescent cell antiapoptotic pathway (SCAP). Quercetin is considered to be the most effective agent in eliminating senescent BM-MSCs [160]. Xing’s research group has created two bone-specific drug delivery systems: bone-mimetic peptide-6-modified liposomes and matrix metalloproteinase (MMP)-responsive hydrogels. These systems efficiently encapsulate quercetin, facilitating the targeted elimination of senescent BM-MSCs within bone tissue, stimulating bone regeneration in SOP murine models, and minimizing adverse effects on other organs [4,161]. However, senolytic strategies may be effective when senescent cells are scarce. As individuals age, the prevalence of senescent cells in their tissues rises, and their removal at this stage can cause significant tissue damage and impaired organ function [162].

Senomorphic agents represent another treatment strategy that mainly targets SASP. Classic senomorphic drugs, such as JAK inhibitors (JAKi), can rejuvenate BM-MSCs in the bone marrow microenvironment without killing senescent cells; this is achieved through the attenuation of SASP signaling, and it effectively promoted bone formation in aged osteoporotic mice [12]. However, senomorphic strategies may indiscriminately eliminate SASP factors that are beneficial for the organism. In addition, as senescent cells persist in the body, the continuous use of SASP inhibitors may be required, potentially causing more side effects than senolytics, which appear to be effective even with intermittent administration [163].

**Table 2:** Summary of the protective effect of drugs on SOP by promoting the rejuvenation of senescent BM-MSCs.

Drugs or Regulators	Setting		Intervention	Target	Mechanism	References
DOPs	In vitro	Senescent human BM-MSCs	400 µg/mL	Nrf2	Antioxidant	[155]
	In vivo	15-month-old mice	150 mg/kg, once daily for 3 months			
PQQ	In vitro	Senescent mouse BM-MSCs	Isolated from PQQ-treated 18-month-old mice	MCM3-Keap1-Nrf2	Antioxidant	[154]
	In vivo	12-month-old male mice (C57BL/6)	4 mg/kg diet for 6 months			
RESV	In vitro	Senescent mouse BM-MSCs	10 μM	Mitofilin	Mitochondrial function	[48]
	In vivo	SAMP6 mice	100 mg/kg intraperitoneally once every other day for 2 months			
Curculigoside	In vitro	Senescent mice BM-MSCs	100 μM	TAZ	Antioxidant	[156]
	In vivo	16-month-old mice (C57BL/6)	100 mg/kg, once daily for 2 months			
Apocynin	In vitro	Senescent rat BM-MSCs	100 μM	NADPH oxidases	Antioxidant	[164]
	In vivo	22-month-old rats (SD) SAMP6 mice	0.1 mg/kg/day, through intraperitoneal injection three times per week for 3 months			
Desferal^®^	In vitro	Senescent rat BM-MSCs	Isolated from 12- month-old rats: 60 mg/kg, once a day for ten consecutive days	HIF-1α	Intercellular communication	[9]
	In vivo	12- month-old rats (SD)	60 mg/kg, three times a week for 8 weeks			
Alpha-ketoglutarate (αKG)	In vitro	Senescent mouse BM-MSCs	2 mM	H3K9me3; H3K27me3	Epigenetic regulation	[152]
	In vivo	18-month- old mice and 24-month-old rats	0.75% αKG in drinking water			
1,25(OH)2D3	In vitro	Senescent human BM-MSCs	100 nM	H3K27me3	Epigenetic regulation;	[153]
	In vivo	12-month-old mice	0.1 μg/kg, thrice weekly for 6 months			
Tetramethylpyrazine (TMP)	In vitro	Senescent mouse BM-MSCs	50 μM	H3k27me3; AMPK—mTOR—HIF-1α—VEGF signaling pathway	Epigenetic regulation; nutrient-sensing network; anti-inflammatory	[157]
	In vivo	20-month-old male mice	10 μg/kg for 8 weeks			
Melatonin	In vitro	Senescent human BM-MSCs	1 μM	NSD2-H3K36me2; H3K27me3; NF-κB	Epigenetic regulation; anti-inflammatory	[158,159]
	In vivo	18-month-old mice (C57BL/6)	10 mg/kg, twice a week for 10 weeks			
Liposomes decorated with Bone affinity peptide (DSS)6 carry quercetin	In vitro	100 μM H_2_O_2_ for 24 h or 45 g/L D-gal for 48 h induces mice BM-MSCs senescence	20 μM quercetin	bone-targeted delivery of senolytics efficiently eliminates senescent BM-MSCs	Eliminates senescent BM-MSCs	[4]
	In vivo	24-month-old male C57bl/6 mice and senescence-accelerated mouse model induced by doxorubicin	150 μL quercetin nanoliposome suspension			

#### 4.2.2. Extracellular Vesicle Therapy

EVs have been shown to facilitate both short-range and long-range intercellular communication without accumulating in the microvasculature. This property effectively mitigates the risk of embolism associated with direct administration [165]. Given the aforementioned advantages of EVs, they may become a key tool in improving the therapeutic effects of SOP treatment [166].

Multiple studies have indicated that umbilical cord blood extracellular vesicles (UCB-EVs) inhibit the senescent phenotype of BM-MSCs and increase their self-renewal capacity and the telomere length through cytokines such as TGF-β, EGF, FGF, VEGF, IGF, and miR-3960, resulting in good therapeutic effects in animal models of SOP [167,168,169]. Other studies have indicated that small extracellular vesicles derived from human embryonic stem cells (HESC-SEVs) promote the rejuvenation of senescent BM-MSCs through the activation of the Sirtuin and AMPK signaling pathways, effectively mitigating age-related bone loss. In the apoptotic vesicles (apoVs) of young MSCs, it was found that Rab7 could rejuvenate BM-MSCs by activating the autophagy process, thereby alleviating bone loss in aging mice [170].

EV therapy, as a novel cell-free alternative treatment method, offers the advantage of lower immunogenicity; this means that immune rejection reactions are prevented and the EVs’ contents are protected from degradation. However, due to technological limitations, no method currently exists for the efficient and thorough separation and purification of different types of EVs. This restricts the broader application of EVs in clinical trials.

#### 4.2.3. Mechanical Stimulation Therapy

In 1892, the German anatomist and surgeon Julius Wolff proposed Wolff’s law, which states that bone growth, resorption, and remodeling are related to the state of bone loading. This suggests that the rejuvenation of senescent BM-MSCs through mechanical stimulation may represent an innovative, non-drug, and non-invasive strategy to alleviate SOP [171]. For instance, low-magnitude vibration (LMV), administered at 0.3 g and 90 Hz for 30 min daily, was found to suppress the senescence of BM-MSCs through the Sirt1/p53/p21 axis. LMV also upregulated the expression of mechanosensitive miR-378a-3p in senescent BM-MSCs, improving bone structure and biomechanical properties in aged rats [172]. The tensile strain of cells produced through appropriate physical exercise promotes the expression of lncRNA-MEG3 in senescent BM-MSCs and enhances osteogenesis, representing an effective physical strategy to treat SOP [173]. In addition, electrical fields and electrical signals are indispensable physical factors in cellular life activities. P-TENG revitalizes senescent BM-MSCs by activating the mechanosensitive cation channel Piezo1 and increasing the intracellular calcium levels; in this way, it promoted bone formation in aging mice [174,175]. However, due to variations in the treatment parameters and individual responses, mechanical stimulation therapy requires additional investigation in clinical research to optimize its effectiveness.

### 4.3. Safety and Prospects

While the rejuvenation of BM-MSCs holds great promise in the treatment of SOP, it is crucial to address the associated safety concerns. In many cases, cellular senescence serves as a natural barrier against tumorigenesis; thus, the excessive suppression of senescence may heighten the risk of tumor development. For example, the moderate activation of AMPK is beneficial for BM-MSC rejuvenation and may initially exert a tumor-suppressive effect. However, excessive AMPK activation can lead to uncontrolled cell cycling, potentially promoting tumorigenesis [176]. Similarly, while antioxidants support BM-MSCs’ rejuvenation, they also pose potential risks associated with tumorigenicity [177,178]. Research suggests that the improper activation of NRF2 may drive tumor progression by inhibiting the degradation of Bach1 [179,180]. Additionally, other studies indicate that excessive NMN levels could lead to the significant secretion of pro-inflammatory SASP, further increasing the risk of tumor development [181].

Overall, the challenge of effectively promoting BM-MSC rejuvenation while mitigating harmful side effects remains a key focus in this field. Therefore, we propose several potential solutions and future research directions that could aid in addressing this issue.

**Figure 5:**
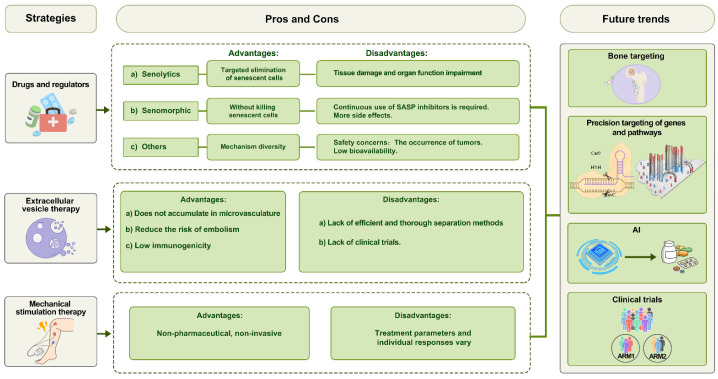
The advantages, limitations, and future trends of various BM-MSC rejuvenation strategies in the treatment of SOP.

The first is **bone targeting**, as bone-specific therapeutic strategies can significantly minimize the risk of adverse effects on other organs. The second is the **precision targeting of genes and pathways**, as different subpopulations of senescent BM-MSCs possess distinct intrinsic programs [182]. By leveraging gene screening technologies such as CRISPR-Cas9 [183], researchers can identify the specific genes driving the senescence processes of each subpopulation, paving the way for strategies that minimize harmful side effects during BM-MSC rejuvenation. The third is **artificial intelligence (AI)**; recent research has driven the development of numerous virtual drug screening platforms, such as AlphaFold 3 [184]. AI technology will enhance the precision of dosage control and target selection, optimizing treatment efficacy while minimizing the risks [185,186]. ***Finally*****, clinical trials** are crucial, as most BM-MSC rejuvenation studies are still in the preclinical stage. Comprehensive clinical data will be essential to assess the feasibility and safety of BM-MSC rejuvenation for SOP treatment (Figure 5).

## 5. Conclusions

In summary, this article explores the core mechanisms of senescent BM-MSC rejuvenation and their applications in treating SOP. Looking ahead, integrating bone-targeting strategies, precision gene and pathway modulation, AI-driven approaches, and clinical trials may pave the way for more effective and personalized strategies for SOP prevention and treatment.

## Data Availability

No new data were created or analyzed in this study. Data sharing is not applicable to this article.

## References

[1] Dominici M., Le Blanc K., Mueller I., Slaper-Cortenbach I., Marini F., Krause D., Deans R., Keating A., Prockop D., Horwitz E. (2006). Minimal criteria for defining multipotent mesenchymal stromal cells. The International Society for Cellular Therapy position statement. Cytotherapy.

[2] Calcinotto A., Kohli J., Zagato E., Pellegrini L., Demaria M., Alimonti A. (2019). Cellular Senescence: Aging, Cancer, and Injury. Physiol. Rev..

[3] Chaib S., Tchkonia T., Kirkland J.L. (2022). Cellular senescence and senolytics: The path to the clinic. Nat. Med..

[4] Xing X., Tang Q., Zou J., Huang H., Yang J., Gao X., Xu X., Ma S., Li M., Liang C. (2023). Bone-targeted delivery of senolytics to eliminate senescent cells increases bone formation in senile osteoporosis. Acta Biomater..

[5] Basisty N., Kale A., Jeon O.H., Kuehnemann C., Payne T., Rao C., Holtz A., Shah S., Sharma V., Ferrucci L. (2020). A proteomic atlas of senescence-associated secretomes for aging biomarker development. PLoS Biol..

[6] Campisi J., Kapahi P., Lithgow G.J., Melov S., Newman J.C., Verdin E. (2019). From discoveries in ageing research to therapeutics for healthy ageing. Nature.

[7] Li J., Huang Y., Sun H., Yang L. (2023). Mechanism of mesenchymal stem cells and exosomes in the treatment of age-related diseases. Front. Immunol..

[8] Zhang L., Pitcher L.E., Yousefzadeh M.J., Niedernhofer L.J., Robbins P.D., Zhu Y. (2022). Cellular senescence: A key therapeutic target in aging and diseases. J. Clin. Investig..

[9] Yi L., Ju Y., He Y., Yin X., Xu Y., Weng T. (2021). Intraperitoneal injection of Desferal^®^ alleviated the age-related bone loss and senescence of bone marrow stromal cells in rats. Stem Cell Res. Ther..

[10] Alicka M., Kornicka-Garbowska K., Kucharczyk K., Kępska M., Röcken M., Marycz K. (2020). Age-dependent impairment of adipose-derived stem cells isolated from horses. Stem Cell Res. Ther..

[11] Tang B., Chen Y., Zhao P., Yan W., Huang X., Jiang W., Sun M., Zhang H., Xiang D., Chen T. (2023). MiR-601-induced BMSCs senescence accelerates steroid-induced osteonecrosis of the femoral head progression by targeting SIRT1. Cell Mol. Life Sci..

[12] Farr J.N., Xu M., Weivoda M.M., Monroe D.G., Fraser D.G., Onken J.L., Negley B.A., Sfeir J.G., Ogrodnik M.B., Hachfeld C.M. (2017). Targeting cellular senescence prevents age-related bone loss in mice. Nat. Med..

[13] Guo Y., Jia X., Cui Y., Song Y., Wang S., Geng Y., Li R., Gao W., Fu D. (2021). Sirt3-mediated mitophagy regulates AGEs-induced BMSCs senescence and senile osteoporosis. Redox Biol..

[14] Wang C., Yang K., Liu X., Wang S., Song M., Belmonte J.C.I., Qu J., Liu G.H., Zhang W. (2023). MAVS Antagonizes Human Stem Cell Senescence as a Mitochondrial Stabilizer. Research.

[15] Yang Y.K., Ogando C.R., Wang See C., Chang T.Y., Barabino G.A. (2018). Changes in phenotype and differentiation potential of human mesenchymal stem cells aging in vitro. Stem Cell Res. Ther..

[16] Marklein R.A., Lo Surdo J.L., Bellayr I.H., Godil S.A., Puri R.K., Bauer S.R. (2016). High Content Imaging of Early Morphological Signatures Predicts Long Term Mineralization Capacity of Human Mesenchymal Stem Cells upon Osteogenic Induction. Stem Cells.

[17] Watts G. (2011). Leonard Hayflick and the limits of ageing. Lancet.

[18] Ding J., Zhang R., Li H., Ji Q., Cheng X., Thorne R.F., Hondermarck H., Liu X., Shen C. (2021). ASIC1 and ASIC3 mediate cellular senescence of human nucleus pulposus mesenchymal stem cells during intervertebral disc degeneration. Aging.

[19] Stenderup K., Justesen J., Clausen C., Kassem M. (2003). Aging is associated with decreased maximal life span and accelerated senescence of bone marrow stromal cells. Bone.

[20] Yu B., Wang C.Y. (2016). Osteoporosis: The Result of an ’Aged’ Bone Microenvironment. Trends Mol. Med..

[21] Le Clainche C., Carlier M.F. (2008). Regulation of actin assembly associated with protrusion and adhesion in cell migration. Physiol. Rev..

[22] Sepúlveda J.C., Tomé M., Fernández M.E., Delgado M., Campisi J., Bernad A., González M.A. (2014). Cell senescence abrogates the therapeutic potential of human mesenchymal stem cells in the lethal endotoxemia model. Stem Cells.

[23] Eisa N.H., Sudharsan P.T., Herrero S.M., Herberg S.A., Volkman B.F., Aguilar-Pérez A., Kondrikov D., Elmansi A.M., Reitman C., Shi X. (2021). Age-associated changes in microRNAs affect the differentiation potential of human mesenchymal stem cells: Novel role of miR-29b-1-5p expression. Bone.

[24] Li H., Liu P., Xu S., Li Y., Dekker J.D., Li B., Fan Y., Zhang Z., Hong Y., Yang G. (2017). FOXP1 controls mesenchymal stem cell commitment and senescence during skeletal aging. J. Clin. Investig..

[25] Moerman E.J., Teng K., Lipschitz D.A., Lecka-Czernik B. (2004). Aging activates adipogenic and suppresses osteogenic programs in mesenchymal marrow stroma/stem cells: The role of PPAR-gamma2 transcription factor and TGF-beta/BMP signaling pathways. Aging cell.

[26] Cheng M., Yuan W., Moshaverinia A., Yu B. (2023). Rejuvenation of Mesenchymal Stem Cells to Ameliorate Skeletal Aging. Cells.

[27] Weng Z., Wang Y., Ouchi T., Liu H., Qiao X., Wu C., Zhao Z., Li L., Li B. (2022). Mesenchymal Stem/Stromal Cell Senescence: Hallmarks, Mechanisms, and Combating Strategies. Stem Cells Transl. Med..

[28] Yang Z.X., Mao G.X., Zhang J., Wen X.L., Jia B.B., Bao Y.Z., Lv X.L., Wang Y.Z., Wang G.F. (2017). IFN-γ induces senescence-like characteristics in mouse bone marrow mesenchymal stem cells. Adv. Clin. Exp. Med..

[29] Ben-Ami E., Miller A., Berrih-Aknin S. (2014). T cells from autoimmune patients display reduced sensitivity to immunoregulation by mesenchymal stem cells: Role of IL-2. Autoimmun. Rev..

[30] Turinetto V., Vitale E., Giachino C. (2016). Senescence in Human Mesenchymal Stem Cells: Functional Changes and Implications in Stem Cell-Based Therapy. Int. J. Mol. Sci..

[31] Massaro F., Corrillon F., Stamatopoulos B., Dubois N., Ruer A., Meuleman N., Bron D., Lagneaux L. (2023). Age-related changes in human bone marrow mesenchymal stromal cells: Morphology, gene expression profile, immunomodulatory activity and miRNA expression. Front. Immunol..

[32] Friedman J.R., Nunnari J. (2014). Mitochondrial form and function. Nature.

[33] Miwa S., Kashyap S., Chini E., von Zglinicki T. (2022). Mitochondrial dysfunction in cell senescence and aging. J. Clin. Investig..

[34] Zorov D.B., Juhaszova M., Sollott S.J. (2014). Mitochondrial reactive oxygen species (ROS) and ROS-induced ROS release. Physiol. Rev..

[35] Hernansanz-Agustín P., Choya-Foces C., Carregal-Romero S., Ramos E., Oliva T., Villa-Piña T., Moreno L., Izquierdo-Álvarez A., Cabrera-García J.D., Cortés A. (2020). Na(+) controls hypoxic signalling by the mitochondrial respiratory chain. Nature.

[36] Green D.R., Galluzzi L., Kroemer G. (2011). Mitochondria and the autophagy-inflammation-cell death axis in organismal aging. Science.

[37] Phua Q.H., Ng S.Y., Soh B.S. (2024). Mitochondria: A Potential Rejuvenation Tool against Aging. Aging Dis..

[38] Li Q., Gao Z., Chen Y., Guan M.X. (2017). The role of mitochondria in osteogenic, adipogenic and chondrogenic differentiation of mesenchymal stem cells. Protein Cell.

[39] Wang Y., Liu Y., Chen E., Pan Z. (2020). The role of mitochondrial dysfunction in mesenchymal stem cell senescence. Cell Tissue Res..

[40] Ren L., Chen X., Chen X., Li J., Cheng B., Xia J. (2020). Mitochondrial Dynamics: Fission and Fusion in Fate Determination of Mesenchymal Stem Cells. Front. Cell Dev. Biol..

[41] Li X., Hong Y., He H., Jiang G., You W., Liang X., Fu Q., Han S., Lian Q., Zhang Y. (2019). FGF21 Mediates Mesenchymal Stem Cell Senescence via Regulation of Mitochondrial Dynamics. Oxid. Med. Cell Longev..

[42] Suh J., Kim N.K., Shim W., Lee S.H., Kim H.J., Moon E., Sesaki H., Jang J.H., Kim J.E., Lee Y.S. (2023). Mitochondrial fragmentation and donut formation enhance mitochondrial secretion to promote osteogenesis. Cell Metab..

[43] Gan X., Huang S., Yu Q., Yu H., Yan S.S. (2015). Blockade of Drp1 rescues oxidative stress-induced osteoblast dysfunction. Biochem. Biophys. Res. Commun..

[44] Kleele T., Rey T., Winter J., Zaganelli S., Mahecic D., Perreten Lambert H., Ruberto F.P., Nemir M., Wai T., Pedrazzini T. (2021). Distinct fission signatures predict mitochondrial degradation or biogenesis. Nature.

[45] Liu J., Bao X., Huang J., Chen R., Tan Y., Zhang Z., Xiao B., Kong F., Gu C., Du J. (2024). TMEM135 maintains the equilibrium of osteogenesis and adipogenesis by regulating mitochondrial dynamics. Metabolism.

[46] Seth R.B., Sun L., Ea C.K., Chen Z.J. (2005). Identification and characterization of MAVS, a mitochondrial antiviral signaling protein that activates NF-kappaB and IRF 3. Cell.

[47] Sikora M., Śmieszek A., Pielok A., Marycz K. (2023). MiR-21-5p regulates the dynamic of mitochondria network and rejuvenates the senile phenotype of bone marrow stromal cells (BMSCs) isolated from osteoporotic SAM/P6 mice. Stem Cell Res. Ther..

[48] Lv Y.J., Yang Y., Sui B.D., Hu C.H., Zhao P., Liao L., Chen J., Zhang L.Q., Yang T.T., Zhang S.F. (2018). Resveratrol counteracts bone loss via mitofilin-mediated osteogenic improvement of mesenchymal stem cells in senescence-accelerated mice. Theranostics.

[49] Wang S., Deng Z., Ma Y., Jin J., Qi F., Li S., Liu C., Lyu F.J., Zheng Q. (2020). The Role of Autophagy and Mitophagy in Bone Metabolic Disorders. Int. J. Biol. Sci..

[50] Zhang F., Peng W., Zhang J., Dong W., Wu J., Wang T., Xie Z. (2020). P53 and Parkin co-regulate mitophagy in bone marrow mesenchymal stem cells to promote the repair of early steroid-induced osteonecrosis of the femoral head. Cell Death Dis..

[51] Fan P., Yu X.Y., Chen C.H., Gao J.W., Xu Y.Z., Xie X.H., Wang Y.T. (2022). Parkin-mediated mitophagy protects against TNF-α-induced stress in bone marrow mesenchymal stem cells. Exp. Gerontol..

[52] Zhao W., Zhao B., Meng X., Li B., Wang Y., Yu F., Fu C., Yu X., Li X., Dai C. (2024). The regulation of MFG-E8 on the mitophagy in diabetic sarcopenia via the HSPA1L-Parkin pathway and the effect of D-pinitol. J. Cachexia Sarcopenia Muscle.

[53] Lee J.H., Yoon Y.M., Song K.H., Noh H., Lee S.H. (2020). Melatonin suppresses senescence-derived mitochondrial dysfunction in mesenchymal stem cells via the HSPA1L-mitophagy pathway. Aging Cell.

[54] Terešak P., Lapao A., Subic N., Boya P., Elazar Z., Simonsen A. (2022). Regulation of PRKN-independent mitophagy. Autophagy.

[55] Zhang H., Xu R., Li B., Xin Z., Ling Z., Zhu W., Li X., Zhang P., Fu Y., Chen J. (2022). LncRNA NEAT1 controls the lineage fates of BMSCs during skeletal aging by impairing mitochondrial function and pluripotency maintenance. Cell Death Differ..

[56] Herzig S., Shaw R.J. (2018). AMPK: Guardian of metabolism and mitochondrial homeostasis. Nat. Rev. Mol. Cell Biol..

[57] Yang Q., Zou Y., Wei X., Ye P., Wu Y., Ai H., Zhang Z., Tan J., Zhou J., Yang Y. (2023). PTP1B knockdown alleviates BMSCs senescence via activating AMPK-mediated mitophagy and promotes osteogenesis in senile osteoporosis. Biochim. Biophys. Acta Mol. Basis Dis..

[58] Liu F., Yuan Y., Bai L., Yuan L., Li L., Liu J., Chen Y., Lu Y., Cheng J., Zhang J. (2021). LRRc17 controls BMSC senescence via mitophagy and inhibits the therapeutic effect of BMSCs on ovariectomy-induced bone loss. Redox Biol..

[59] Zhao B., Peng Q., Wang D., Zhou R., Wang R., Zhu Y., Qi S. (2022). Leonurine Protects Bone Mesenchymal Stem Cells from Oxidative Stress by Activating Mitophagy through PI3K/Akt/mTOR Pathway. Cells.

[60] Zhou T., Yan Y., Zhao C., Xu Y., Wang Q., Xu N. (2019). Resveratrol improves osteogenic differentiation of senescent bone mesenchymal stem cells through inhibiting endogenous reactive oxygen species production via AMPK activation. Redox Rep..

[61] Tao H., Zhu P., Xia W., Chu M., Chen K., Wang Q., Gu Y., Lu X., Bai J., Geng D. (2024). The Emerging Role of the Mitochondrial Respiratory Chain in Skeletal Aging. Aging Dis..

[62] Wang H., Sun Y., Pi C., Yu X., Gao X., Zhang C., Sun H., Zhang H., Shi Y., He X. (2022). Nicotinamide Mononucleotide Supplementation Improves Mitochondrial Dysfunction and Rescues Cellular Senescence by NAD(+)/Sirt3 Pathway in Mesenchymal Stem Cells. Int. J. Mol. Sci..

[63] He Y., Ji Q., Wu Z., Cai Y., Yin J., Zhang Y., Zhang S., Liu X., Zhang W., Liu G.H. (2023). 4E-BP1 counteracts human mesenchymal stem cell senescence via maintaining mitochondrial homeostasis. Protein Cell.

[64] Yang J., Griffin A., Qiang Z., Ren J. (2022). Organelle-targeted therapies: A comprehensive review on system design for enabling precision oncology. Signal Transduct. Target. Ther..

[65] Klier P.E.Z., Martin J.G., Miller E.W. (2021). Imaging Reversible Mitochondrial Membrane Potential Dynamics with a Masked Rhodamine Voltage Reporter. J. Am. Chem. Soc..

[66] Kwon H.J., Cha M.Y., Kim D., Kim D.K., Soh M., Shin K., Hyeon T., Mook-Jung I. (2016). Mitochondria-Targeting Ceria Nanoparticles as Antioxidants for Alzheimer’s Disease. ACS Nano.

[67] Gu C., Zhou Q., Hu X., Ge X., Hou M., Wang W., Liu H., Shi Q., Xu Y., Zhu X. (2024). Melatonin rescues the mitochondrial function of bone marrow-derived mesenchymal stem cells and improves the repair of osteoporotic bone defect in ovariectomized rats. J. Pineal Res..

[68] Chen W., Lv N., Liu H., Gu C., Zhou X., Qin W., Chen A.C., Chen L., Yang H., Chen X. (2022). Melatonin Improves the Resistance of Oxidative Stress-Induced Cellular Senescence in Osteoporotic Bone Marrow Mesenchymal Stem Cells. Oxid. Med. Cell Longev..

[69] Lee J., Kim Y., Liu T., Hwang Y.J., Hyeon S.J., Im H., Lee K., Alvarez V.E., McKee A.C., Um S.J. (2018). SIRT3 deregulation is linked to mitochondrial dysfunction in Alzheimer’s disease. Aging Cell.

[70] Fang J., Zhao X., Li S., Xing X., Wang H., Lazarovici P., Zheng W. (2019). Protective mechanism of artemisinin on rat bone marrow-derived mesenchymal stem cells against apoptosis induced by hydrogen peroxide via activation of c-Raf-Erk1/2-p90(rsk)-CREB pathway. Stem Cell Res. Ther..

[71] Davinelli S., Medoro A., Intrieri M., Saso L., Scapagnini G., Kang J.X. (2022). Targeting NRF2-KEAP1 axis by Omega-3 fatty acids and their derivatives: Emerging opportunities against aging and diseases. Free Radic. Biol. Med..

[72] Atalay Ekiner S., Gęgotek A., Skrzydlewska E. (2022). The molecular activity of cannabidiol in the regulation of Nrf2 system interacting with NF-κB pathway under oxidative stress. Redox Biol..

[73] Kou Y., Rong X., Tang R., Zhang Y., Yang P., Liu H., Ma W., Li M. (2023). Eldecalcitol prevented OVX-induced osteoporosis through inhibiting BMSCs senescence by regulating the SIRT1-Nrf2 signal. Front. Pharmacol..

[74] Compston J.E., McClung M.R., Leslie W.D. (2019). Osteoporosis. Lancet.

[75] Widjaja A.A., Lim W.W., Viswanathan S., Chothani S., Corden B., Dasan C.M., Goh J.W.T., Lim R., Singh B.K., Tan J. (2024). Inhibition of IL-11 signalling extends mammalian healthspan and lifespan. Nature.

[76] Gulen M.F., Samson N., Keller A., Schwabenland M., Liu C., Glück S., Thacker V.V., Favre L., Mangeat B., Kroese L.J. (2023). cGAS-STING drives ageing-related inflammation and neurodegeneration. Nature.

[77] Wang Z., Li L., Gu W., Mao Y., Wang T. (2021). Resveratrol Reverses Osteogenic Decline of Bone Marrow Mesenchymal Stem Cells Via Upregulation of YAP Expression in Inflammatory Environment. Stem Cells Dev..

[78] Wu M., Luo Q., Nie R., Yang X., Tang Z., Chen H. (2021). Potential implications of polyphenols on aging considering oxidative stress, inflammation, autophagy, and gut microbiota. Crit. Rev. Food Sci. Nutr..

[79] De la Fuente M., Miquel J. (2009). An update of the oxidation-inflammation theory of aging: The involvement of the immune system in oxi-inflamm-aging. Curr. Pharm. Des..

[80] Chung H.Y., Cesari M., Anton S., Marzetti E., Giovannini S., Seo A.Y., Carter C., Yu B.P., Leeuwenburgh C. (2009). Molecular inflammation: Underpinnings of aging and age-related diseases. Ageing Res. Rev..

[81] Hu M., Xing L., Zhang L., Liu F., Wang S., Xie Y., Wang J., Jiang H., Guo J., Li X. (2022). NAP1L2 drives mesenchymal stem cell senescence and suppresses osteogenic differentiation. Aging Cell.

[82] Deng Y., Lu J., Li W., Wu A., Zhang X., Tong W., Ho K.K., Qin L., Song H., Mak K.K. (2018). Reciprocal inhibition of YAP/TAZ and NF-κB regulates osteoarthritic cartilage degradation. Nat. Commun..

[83] Yu B., Huo L., Liu Y., Deng P., Szymanski J., Li J., Luo X., Hong C., Lin J., Wang C.Y. (2018). PGC-1α Controls Skeletal Stem Cell Fate and Bone-Fat Balance in Osteoporosis and Skeletal Aging by Inducing TAZ. Cell Stem Cell.

[84] Zhao K., Zhou T., Yang J., Li Y., Qin J., Wang S., Li D., Chen J., Zheng W. (2023). Lutein shows a protective effect against the aging of mesenchymal stem cells by downregulating inflammation. Int. Immunopharmacol..

[85] Amorim J.A., Coppotelli G., Rolo A.P., Palmeira C.M., Ross J.M., Sinclair D.A. (2022). Mitochondrial and metabolic dysfunction in ageing and age-related diseases. Nat. Rev. Endocrinol..

[86] Duran-Ortiz S., List E.O., Ikeno Y., Young J., Basu R., Bell S., McHugh T., Funk K., Mathes S., Qian Y. (2021). Growth hormone receptor gene disruption in mature-adult mice improves male insulin sensitivity and extends female lifespan. Aging Cell.

[87] Xian L., Wu X., Pang L., Lou M., Rosen C.J., Qiu T., Crane J., Frassica F., Zhang L., Rodriguez J.P. (2012). Matrix IGF-1 maintains bone mass by activation of mTOR in mesenchymal stem cells. Nat. Med..

[88] Chen C.Y., Tseng K.Y., Lai Y.L., Chen Y.S., Lin F.H., Lin S. (2017). Overexpression of Insulin-Like Growth Factor 1 Enhanced the Osteogenic Capability of Aging Bone Marrow Mesenchymal Stem Cells. Theranostics.

[89] Chu Q., Liu F., He Y., Jiang X., Cai Y., Wu Z., Yan K., Geng L., Zhang Y., Feng H. (2022). mTORC2/RICTOR exerts differential levels of metabolic control in human embryonic, mesenchymal and neural stem cells. Protein Cell.

[90] Meng D., Frank A.R., Jewell J.L. (2018). mTOR signaling in stem and progenitor cells. Development.

[91] Yang M., Teng S., Ma C., Yu Y., Wang P., Yi C. (2018). Ascorbic acid inhibits senescence in mesenchymal stem cells through ROS and AKT/mTOR signaling. Cytotechnology.

[92] Liu G., Li X., Yang F., Qi J., Shang L., Zhang H., Li S., Xu F., Li L., Yu H. (2023). C-Phycocyanin Ameliorates the Senescence of Mesenchymal Stem Cells through ZDHHC5-Mediated Autophagy via PI3K/AKT/mTOR Pathway. Aging Dis..

[93] O’Callaghan C., Vassilopoulos A. (2017). Sirtuins at the crossroads of stemness, aging, and cancer. Aging Cell.

[94] Oh J., Lee Y.D., Wagers A.J. (2014). Stem cell aging: Mechanisms, regulators and therapeutic opportunities. Nat. Med..

[95] Denu R.A. (2017). SIRT3 Enhances Mesenchymal Stem Cell Longevity and Differentiation. Oxid. Med. Cell Longev..

[96] Wang H., Hu Z., Wu J., Mei Y., Zhang Q., Zhang H., Miao D., Sun W. (2019). Sirt1 Promotes Osteogenic Differentiation and Increases Alveolar Bone Mass via Bmi1 Activation in Mice. J. Bone Miner. Res..

[97] Brunet A., Sweeney L.B., Sturgill J.F., Chua K.F., Greer P.L., Lin Y., Tran H., Ross S.E., Mostoslavsky R., Cohen H.Y. (2004). Stress-dependent regulation of FOXO transcription factors by the SIRT1 deacetylase. Science.

[98] Sun W., Qiao W., Zhou B., Hu Z., Yan Q., Wu J., Wang R., Zhang Q., Miao D. (2018). Overexpression of Sirt1 in mesenchymal stem cells protects against bone loss in mice by FOXO3a deacetylation and oxidative stress inhibition. Metabolism.

[99] Chen X., Yan J., He F., Zhong D., Yang H., Pei M., Luo Z.P. (2018). Mechanical stretch induces antioxidant responses and osteogenic differentiation in human mesenchymal stem cells through activation of the AMPK-SIRT1 signaling pathway. Free Radic. Biol. Med..

[100] Chen H., Liu X., Zhu W., Chen H., Hu X., Jiang Z., Xu Y., Wang L., Zhou Y., Chen P. (2014). SIRT1 ameliorates age-related senescence of mesenchymal stem cells via modulating telomere shelterin. Front. Aging Neurosci..

[101] Liu F., Yuan L., Li L., Yang J., Liu J., Chen Y., Zhang J., Lu Y., Yuan Y., Cheng J. (2023). S-sulfhydration of SIRT3 combats BMSC senescence and ameliorates osteoporosis via stabilizing heterochromatic and mitochondrial homeostasis. Pharmacol. Res..

[102] Liao C.Y., Kennedy B.K. (2016). SIRT6, oxidative stress, and aging. Cell Res..

[103] Pan H., Guan D., Liu X., Li J., Wang L., Wu J., Zhou J., Zhang W., Ren R., Zhang W. (2016). SIRT6 safeguards human mesenchymal stem cells from oxidative stress by coactivating NRF2. Cell Res..

[104] Zhong L., D’Urso A., Toiber D., Sebastian C., Henry R.E., Vadysirisack D.D., Guimaraes A., Marinelli B., Wikstrom J.D., Nir T. (2010). The histone deacetylase Sirt6 regulates glucose homeostasis via Hif1alpha. Cell.

[105] Kaiser A., Schmidt M., Huber O., Frietsch J.J., Scholl S., Heidel F.H., Hochhaus A., Müller J.P., Ernst T. (2020). SIRT7: An influence factor in healthy aging and the development of age-dependent myeloid stem-cell disorders. Leukemia.

[106] Lagunas-Rangel F.A. (2022). SIRT7 in the aging process. Cell Mol. Life Sci..

[107] Bi S., Liu Z., Wu Z., Wang Z., Liu X., Wang S., Ren J., Yao Y., Zhang W., Song M. (2020). SIRT7 antagonizes human stem cell aging as a heterochromatin stabilizer. Protein Cell.

[108] Jadeja R.N., Powell F.L., Jones M.A., Fuller J., Joseph E., Thounaojam M.C., Bartoli M., Martin P.M. (2018). Loss of NAMPT in aging retinal pigment epithelium reduces NAD(+) availability and promotes cellular senescence. Aging.

[109] Schmeisser K., Mansfeld J., Kuhlow D., Weimer S., Priebe S., Heiland I., Birringer M., Groth M., Segref A., Kanfi Y. (2013). Role of sirtuins in lifespan regulation is linked to methylation of nicotinamide. Nat. Chem. Biol..

[110] Song J., Li J., Yang F., Ning G., Zhen L., Wu L., Zheng Y., Zhang Q., Lin D., Xie C. (2019). Nicotinamide mononucleotide promotes osteogenesis and reduces adipogenesis by regulating mesenchymal stromal cells via the SIRT1 pathway in aged bone marrow. Cell Death Dis..

[111] Diao Z., Ji Q., Wu Z., Zhang W., Cai Y., Wang Z., Hu J., Liu Z., Wang Q., Bi S. (2021). SIRT3 consolidates heterochromatin and counteracts senescence. Nucleic Acids Res..

[112] Lu J.Y., Lin Y.Y., Sheu J.C., Wu J.T., Lee F.J., Chen Y., Lin M.I., Chiang F.T., Tai T.Y., Berger S.L. (2011). Acetylation of yeast AMPK controls intrinsic aging independently of caloric restriction. Cell.

[113] Steinberg G.R., Kemp B.E. (2009). AMPK in Health and Disease. Physiol. Rev..

[114] Xia W., Zhang F., Xie C., Jiang M., Hou M. (2015). Macrophage migration inhibitory factor confers resistance to senescence through CD74-dependent AMPK-FOXO3a signaling in mesenchymal stem cells. Stem Cell Res. Ther..

[115] Li Q., Zhu Z., Wang C., Cai L., Lu J., Wang Y., Xu J., Su Z., Zheng W., Chen X. (2018). CTRP9 ameliorates cellular senescence via PGC-1α/AMPK signaling in mesenchymal stem cells. Int. J. Mol. Med..

[116] Zaki E.S., Sayed R.H., Saad M.A., El-Yamany M.F. (2023). Roflumilast ameliorates ovariectomy-induced depressive-like behavior in rats via activation of AMPK/mTOR/ULK1-dependent autophagy pathway. Life Sci..

[117] Leprivier G., Remke M., Rotblat B., Dubuc A., Mateo A.R., Kool M., Agnihotri S., El-Naggar A., Yu B., Somasekharan S.P. (2013). The eEF2 kinase confers resistance to nutrient deprivation by blocking translation elongation. Cell.

[118] Price N.L., Gomes A.P., Ling A.J., Duarte F.V., Martin-Montalvo A., North B.J., Agarwal B., Ye L., Ramadori G., Teodoro J.S. (2012). SIRT1 is required for AMPK activation and the beneficial effects of resveratrol on mitochondrial function. Cell Metab..

[119] Chen H., Liu X., Chen H., Cao J., Zhang L., Hu X., Wang J. (2014). Role of SIRT1 and AMPK in mesenchymal stem cells differentiation. Ageing Res. Rev..

[120] Wang Y., Chen G., Yan J., Chen X., He F., Zhu C., Zhang J., Lin J., Pan G., Yu J. (2018). Upregulation of SIRT1 by Kartogenin Enhances Antioxidant Functions and Promotes Osteogenesis in Human Mesenchymal Stem Cells. Oxid. Med. Cell Longev..

[121] Jiang J.J., Chen S.M., Chen J., Wu L., Ye J.T., Zhang Q. (2022). Serum IGF-1 levels are associated with sarcopenia in elderly men but not in elderly women. Aging Clin. Exp. Res..

[122] Baghdadi M., Nespital T., Monzó C., Deelen J., Grönke S., Partridge L. (2024). Intermittent rapamycin feeding recapitulates some effects of continuous treatment while maintaining lifespan extension. Mol. Metab..

[123] Rubinsztein D.C., Mariño G., Kroemer G. (2011). Autophagy and aging. Cell.

[124] Wang J., Zhang Y., Cao J., Wang Y., Anwar N., Zhang Z., Zhang D., Ma Y., Xiao Y., Xiao L. (2023). The role of autophagy in bone metabolism and clinical significance. Autophagy.

[125] Ma Y., Qi M., An Y., Zhang L., Yang R., Doro D.H., Liu W., Jin Y. (2018). Autophagy controls mesenchymal stem cell properties and senescence during bone aging. Aging Cell.

[126] Bai L., Liu Y., Zhang X., Chen P., Hang R., Xiao Y., Wang J., Liu C. (2023). Osteoporosis remission via an anti-inflammaging effect by icariin activated autophagy. Biomaterials.

[127] Zhang H., Zhao C., Jiang G., Hu B., Zheng H., Hong Y., Cui Z., Shi L., Li X., Lin F. (2021). Apelin Rejuvenates Aged Human Mesenchymal Stem Cells by Regulating Autophagy and Improves Cardiac Protection After Infarction. Front. Cell Dev. Biol..

[128] Liu Z.Z., Hong C.G., Hu W.B., Chen M.L., Duan R., Li H.M., Yue T., Cao J., Wang Z.X., Chen C.Y. (2021). Autophagy receptor OPTN (optineurin) regulates mesenchymal stem cell fate and bone-fat balance during aging by clearing FABP3. Autophagy.

[129] Deng J., Ouyang P., Li W., Zhong L., Gu C., Shen L., Cao S., Yin L., Ren Z., Zuo Z. (2021). Curcumin Alleviates the Senescence of Canine Bone Marrow Mesenchymal Stem Cells during In Vitro Expansion by Activating the Autophagy Pathway. Int. J. Mol. Sci..

[130] Sahu A., Clemens Z.J., Shinde S.N., Sivakumar S., Pius A., Bhatia A., Picciolini S., Carlomagno C., Gualerzi A., Bedoni M. (2021). Regulation of aged skeletal muscle regeneration by circulating extracellular vesicles. Nat. Aging.

[131] Conboy I.M., Conboy M.J., Wagers A.J., Girma E.R., Weissman I.L., Rando T.A. (2005). Rejuvenation of aged progenitor cells by exposure to a young systemic environment. Nature.

[132] Chen X., Luo Y., Zhu Q., Zhang J., Huang H., Kan Y., Li D., Xu M., Liu S., Li J. (2024). Small extracellular vesicles from young plasma reverse age-related functional declines by improving mitochondrial energy metabolism. Nat. Aging.

[133] Khayrullin A., Krishnan P., Martinez-Nater L., Mendhe B., Fulzele S., Liu Y., Mattison J.A., Hamrick M.W. (2019). Very Long-Chain C24:1 Ceramide Is Increased in Serum Extracellular Vesicles with Aging and Can Induce Senescence in Bone-Derived Mesenchymal Stem Cells. Cells.

[134] Zhang Y., Chen C.Y., Liu Y.W., Rao S.S., Tan Y.J., Qian Y.X., Xia K., Huang J., Liu X.X., Hong C.G. (2021). Neuronal Induction of Bone-Fat Imbalance through Osteocyte Neuropeptide Y. Adv. Sci..

[135] Yu D., Zhang S., Ma C., Huang S., Xu L., Liang J., Li H., Fan Q., Liu G., Zhai Z. (2023). CCL3 in the bone marrow microenvironment causes bone loss and bone marrow adiposity in aged mice. JCI Insight.

[136] López-Otín C., Blasco M.A., Partridge L., Serrano M., Kroemer G. (2023). Hallmarks of aging: An expanding universe. Cell.

[137] Li F., Lun D., Liu D., Jia Z., Zhu Z., Liu Z., Li X. (2023). Melatonin activates mitochondrial unfolded protein response to preserve osteogenic potential of senescent BMSCs via upregulating PDI-6. Biochimie.

[138] Yang R., Cao D., Suo J., Zhang L., Mo C., Wang M., Niu N., Yue R., Zou W. (2023). Premature aging of skeletal stem/progenitor cells rather than osteoblasts causes bone loss with decreased mechanosensation. Bone Res..

[139] Stein K.C., Morales-Polanco F., van der Lienden J., Rainbolt T.K., Frydman J. (2022). Ageing exacerbates ribosome pausing to disrupt cotranslational proteostasis. Nature.

[140] Zeng Q., Gong Y., Zhu N., Shi Y., Zhang C., Qin L. (2024). Lipids and lipid metabolism in cellular senescence: Emerging targets for age-related diseases. Ageing Res. Rev..

[141] Zhao H., Ji Q., Wu Z., Wang S., Ren J., Yan K., Wang Z., Hu J., Chu Q., Hu H. (2022). Destabilizing heterochromatin by APOE mediates senescence. Nat. Aging.

[142] Huo S., Tang X., Chen W., Gan D., Guo H., Yao Q., Liao R., Huang T., Wu J., Yang J. (2024). Epigenetic regulations of cellular senescence in osteoporosis. Ageing Res. Rev..

[143] Sun Y., Zhang H., Qiu T., Liao L., Su X. (2023). Epigenetic regulation of mesenchymal stem cell aging through histone modifications. Genes. Dis..

[144] Zhao Y., He J., Qiu T., Zhang H., Liao L., Su X. (2022). Epigenetic therapy targeting bone marrow mesenchymal stem cells for age-related bone diseases. Stem Cell Res. Ther..

[145] Friedenstein A.J., Chailakhjan R.K., Lalykina K.S. (1970). The development of fibroblast colonies in monolayer cultures of guinea-pig bone marrow and spleen cells. Cell Tissue Kinet..

[146] Zhang L., Guan Q., Wang Z., Feng J., Zou J., Gao B. (2023). Consequences of Aging on Bone. Aging Dis..

[147] Huang T., Lu Z., Wang Z., Cheng L., Gao L., Gao J., Zhang N., Geng C.A., Zhao X., Wang H. (2024). Targeting adipocyte ESRRA promotes osteogenesis and vascular formation in adipocyte-rich bone marrow. Nat. Commun..

[148] Wang T., Huang S., He C. (2022). Senescent cells: A therapeutic target for osteoporosis. Cell Prolif..

[149] Yin S., Lin S., Xu J., Yang G., Chen H., Jiang X. (2023). Dominoes with interlocking consequences triggered by zinc: Involvement of microelement-stimulated MSC-derived exosomes in senile osteogenesis and osteoclast dialogue. J. Nanobiotechnol..

[150] Wang Z.X., Luo Z.W., Li F.X., Cao J., Rao S.S., Liu Y.W., Wang Y.Y., Zhu G.Q., Gong J.S., Zou J.T. (2022). Aged bone matrix-derived extracellular vesicles as a messenger for calcification paradox. Nat. Commun..

[151] Zhang X., Cao D., Xu L., Xu Y., Gao Z., Pan Y., Jiang M., Wei Y., Wang L., Liao Y. (2023). Harnessing matrix stiffness to engineer a bone marrow niche for hematopoietic stem cell rejuvenation. Cell Stem Cell.

[152] Wang Y., Deng P., Liu Y., Wu Y., Chen Y., Guo Y., Zhang S., Zheng X., Zhou L., Liu W. (2020). Alpha-ketoglutarate ameliorates age-related osteoporosis via regulating histone methylations. Nat. Commun..

[153] Yang R., Chen J., Zhang J., Qin R., Wang R., Qiu Y., Mao Z., Goltzman D., Miao D. (2020). 1,25-Dihydroxyvitamin D protects against age-related osteoporosis by a novel VDR-Ezh2-p16 signal axis. Aging Cell.

[154] Li J., Zhang J., Xue Q., Liu B., Qin R., Li Y., Qiu Y., Wang R., Goltzman D., Miao D. (2023). Pyrroloquinoline quinone alleviates natural aging-related osteoporosis via a novel MCM3-Keap1-Nrf2 axis-mediated stress response and Fbn1 upregulation. Aging cell.

[155] Peng H., Yang M., Guo Q., Su T., Xiao Y., Xia Z.Y. (2019). Dendrobium officinale polysaccharides regulate age-related lineage commitment between osteogenic and adipogenic differentiation. Cell proliferation.

[156] Wang N., Li Z., Li S., Li Y., Gao L., Bao X., Wang K., Liu C., Xue P., Liu S. (2021). Curculigoside Ameliorates Bone Loss by Influencing Mesenchymal Stem Cell Fate in Aging Mice. Front. Cell Dev. Biol..

[157] Gao B., Lin X., Jing H., Fan J., Ji C., Jie Q., Zheng C., Wang D., Xu X., Hu Y. (2018). Local delivery of tetramethylpyrazine eliminates the senescent phenotype of bone marrow mesenchymal stromal cells and creates an anti-inflammatory and angiogenic environment in aging mice. Aging Cell.

[158] Xie Y., Han N., Li F., Wang L., Liu G., Hu M., Wang S., Wei X., Guo J., Jiang H. (2022). Melatonin enhances osteoblastogenesis of senescent bone marrow stromal cells through NSD2-mediated chromatin remodelling. Clin. Transl. Med..

[159] Li T., Jiang S., Lu C., Yang W., Yang Z., Hu W., Xin Z., Yang Y. (2019). Melatonin: Another avenue for treating osteoporosis?. J. Pineal Res..

[160] Wang Y., Che L., Chen X., He Z., Song D., Yuan Y., Liu C. (2023). Repurpose dasatinib and quercetin: Targeting senescent cells ameliorates postmenopausal osteoporosis and rejuvenates bone regeneration. Bioact. Mater..

[161] Xing X., Huang H., Gao X., Yang J., Tang Q., Xu X., Wu Y., Li M., Liang C., Tan L. (2022). Local Elimination of Senescent Cells Promotes Bone Defect Repair during Aging. ACS Appl. Mater. Interfaces.

[162] Andrew M.R., Leena G., Owen A.O.C., Rudin C., Divis K., Hua X., Yi-Han C., Raffaella G., Andrew K., Wyndham H.W. (2008). Reduction in platelet counts as a mechanistic biomarker and guide for adaptive dose-escalation in phase I studies of the Bcl-2 family inhibitor ABT-263. J. Clin. Oncol..

[163] Suda M., Paul K.H., Tripathi U., Minamino T., Tchkonia T., Kirkland J.L. (2024). Targeting Cell Senescence and Senolytics: Novel Interventions for Age-Related Endocrine Dysfunction. Endocr. Rev..

[164] Sun J., Ming L., Shang F., Shen L., Chen J., Jin Y. (2015). Apocynin suppression of NADPH oxidase reverses the aging process in mesenchymal stem cells to promote osteogenesis and increase bone mass. Sci. Rep..

[165] Tkach M., Théry C. (2016). Communication by Extracellular Vesicles: Where We Are and Where We Need to Go. Cell.

[166] Fang F., Yang J., Wang J., Li T., Wang E., Zhang D., Liu X., Zhou C. (2024). The role and applications of extracellular vesicles in osteoporosis. Bone Res..

[167] Liang M., Liu W., Peng Z., Lv S., Guan Y., An G., Zhang Y., Huang T., Wang Y. (2019). The therapeutic effect of secretome from human umbilical cord-derived mesenchymal stem cells in age-related osteoporosis. Artif. Cells Nanomed. Biotechnol..

[168] Hu Y., Xu R., Chen C.Y., Rao S.S., Xia K., Huang J., Yin H., Wang Z.X., Cao J., Liu Z.Z. (2019). Extracellular vesicles from human umbilical cord blood ameliorate bone loss in senile osteoporotic mice. Metabolism.

[169] Lei Q., Gao F., Liu T., Ren W., Chen L., Cao Y., Chen W., Guo S., Zhang Q., Chen W. (2021). Extracellular vesicles deposit PCNA to rejuvenate aged bone marrow-derived mesenchymal stem cells and slow age-related degeneration. Sci. Transl. Med..

[170] Fangcao L., Zhiqing H., Qianmin O., Jiaqi L., Yijie L., Lan M., Lingping T., Zhengmei L., Xiaoxing K. (2022). Apoptotic vesicles rejuvenate mesenchymal stem cells via Rab7-mediated autolysosome formation and alleviate bone loss in aging mice. Nano Res..

[171] Vico L., Hargens A. (2018). Skeletal changes during and after spaceflight. Nat. Rev. Rheumatol..

[172] Yu X., Zeng Y., Bao M., Wen J., Zhu G., Cao C., He X., Li L. (2020). Low-magnitude vibration induces osteogenic differentiation of bone marrow mesenchymal stem cells via miR-378a-3p/Grb2 pathway to promote bone formation in a rat model of age-related bone loss. Faseb J..

[173] Zhu G., Zeng C., Qian Y., Yuan S., Ye Z., Zhao S., Li R. (2021). Tensile strain promotes osteogenic differentiation of bone marrow mesenchymal stem cells through upregulating lncRNA-MEG3. Histol. Histopathol..

[174] Li G., Zhu Q., Wang B., Luo R., Xiao X., Zhang Y., Ma L., Feng X., Huang J., Sun X. (2021). Rejuvenation of Senescent Bone Marrow Mesenchymal Stromal Cells by Pulsed Triboelectric Stimulation. Adv. Sci..

[175] Wang B., Li G., Zhu Q., Liu W., Ke W., Hua W., Zhou Y., Zeng X., Sun X., Wen Z. (2022). Bone Repairment via Mechanosensation of Piezo1 Using Wearable Pulsed Triboelectric Nanogenerator. Small.

[176] Kishton R.J., Barnes C.E., Nichols A.G., Cohen S., Gerriets V.A., Siska P.J., Macintyre A.N., Goraksha-Hicks P., de Cubas A.A., Liu T. (2016). AMPK Is Essential to Balance Glycolysis and Mitochondrial Metabolism to Control T-ALL Cell Stress and Survival. Cell Metab..

[177] Wang T., Dong Y., Huang Z., Zhang G., Zhao Y., Yao H., Hu J., Tüksammel E., Cai H., Liang N. (2023). Antioxidants stimulate BACH1-dependent tumor angiogenesis. J. Clin. Investig..

[178] Wiel C., Le Gal K., Ibrahim M.X., Jahangir C.A., Kashif M., Yao H., Ziegler D.V., Xu X., Ghosh T., Mondal T. (2019). BACH1 Stabilization by Antioxidants Stimulates Lung Cancer Metastasis. Cell.

[179] Lu W., Cui J., Wang W., Hu Q., Xue Y., Liu X., Gong T., Lu Y., Ma H., Yang X. (2024). PPIA dictates NRF2 stability to promote lung cancer progression. Nat. Commun..

[180] Lignitto L., LeBoeuf S.E., Homer H., Jiang S., Askenazi M., Karakousi T.R., Pass H.I., Bhutkar A.J., Tsirigos A., Ueberheide B. (2019). Nrf2 Activation Promotes Lung Cancer Metastasis by Inhibiting the Degradation of Bach1. Cell.

[181] Nacarelli T., Lau L., Fukumoto T., Zundell J., Fatkhutdinov N., Wu S., Aird K.M., Iwasaki O., Kossenkov A.V., Schultz D. (2019). NAD(+) metabolism governs the proinflammatory senescence-associated secretome. Nat. Cell Biol..

[182] Zhang Z., Schaefer C., Jiang W., Lu Z., Lee J., Sziraki A., Abdulraouf A., Wick B., Haeussler M., Li Z. (2025). A panoramic view of cell population dynamics in mammalian aging. Science.

[183] Ruetz T.J., Pogson A.N., Kashiwagi C.M., Gagnon S.D., Morton B., Sun E.D., Na J., Yeo R.W., Leeman D.S., Morgens D.W. (2024). CRISPR-Cas9 screens reveal regulators of ageing in neural stem cells. Nature.

[184] Abramson J., Adler J., Dunger J., Evans R., Green T., Pritzel A., Ronneberger O., Willmore L., Ballard A.J., Bambrick J. (2024). Accurate structure prediction of biomolecular interactions with AlphaFold 3. Nature.

[185] Mullard A. (2024). When can AI deliver the drug discovery hits?. Nat. Rev. Drug Discov..

[186] Smer-Barreto V., Quintanilla A., Elliott R.J.R., Dawson J.C., Sun J., Campa V.M., Lorente-Macías Á., Unciti-Broceta A., Carragher N.O., Acosta J.C. (2023). Discovery of senolytics using machine learning. Nat. Commun..

